# Plasmatic and myocardial microRNA profiles in patients with Hypertrophic Cardiomyopathy

**DOI:** 10.1002/ctm2.435

**Published:** 2021-07-19

**Authors:** Maria Lombardi, Davide Lazzeroni, Giulia Benedetti, Gloria Bertoli, Dejan Lazarevic, Michela Riba, Francesco De Cobelli, Ornella Rimoldi, Giulia d'Amati, Iacopo Olivotto, Chiara Foglieni, Paolo Camici

**Affiliations:** ^1^ Cardiovascular Research Center IRCCS San Raffaele Scientific Institute Milan Italy; ^2^ Department of Radiology IRCCS San Raffaele Scientific Institute Milan Italy; ^3^ Institute of Molecular Bioimaging and Physiology National Research Council (IBFM‐CNR) Segrate‐Milan Italy; ^4^ Center for Omics Sciences IRCCS San Raffaele Hospital Milan Italy; ^5^ Department of Radiological Oncological and Pathological Sciences Sapienza University of Rome and Policlinico Umberto I Rome Italy; ^6^ Cardiomyopathy Unit Careggi University Hospital Florence Italy; ^7^ Faculty of Medicine and Surgery Vita‐Salute University via Olgettina, 58 Milan Italy

Dear Editor,

MicroRNAs (miRs) have emerged as cardiovascular biomarkers and myocardial regulators with diagnostic and therapeutic potential.^1^, ^2^ However, the miR profile of patients with hypertrophic cardiomyopathy (HCM) and miRs role in this genetic disease with heterogeneous phenotype^3^ are incompletely determined.

Here a multi‐step strategy provided evidence of differentially expressed miRs (DEmiRs) in plasma and myocardial tissue from HCM (Supplementary Materials: *Materials and Methods)*.

In the first step, a profile of 1128 expressed mature miRs was identified (Figure 1) in 25 plasma samples from a cohort of 36 HCM (Figure S1, Tables S1 and S2) and healthy individuals (CTRL, *n* = 11) by next generation sequencing (NGS). Principal component analysis of NGS data did not clearly define a separation between HCM and CTRL, while a significant differential expression was found in a subset of 139 plasma miRs. Twenty‐eight of them were DEmiRs significant both for *p* value and false discovery rate (Figures 1B and 1C, Tables S3–S4), and demonstrated 1626 putative targets by *in silico* search with miRTargetLink Human engine. Among them, 50 target genes were interconnected with 13 DEmiRs by “strong” experimental methods like reporter gene assay (Figure S2A), corresponded to a complex protein network (Figure S2B) with associated functions/pathways (Supplemental_Enrichment).

**FIGURE 1:**
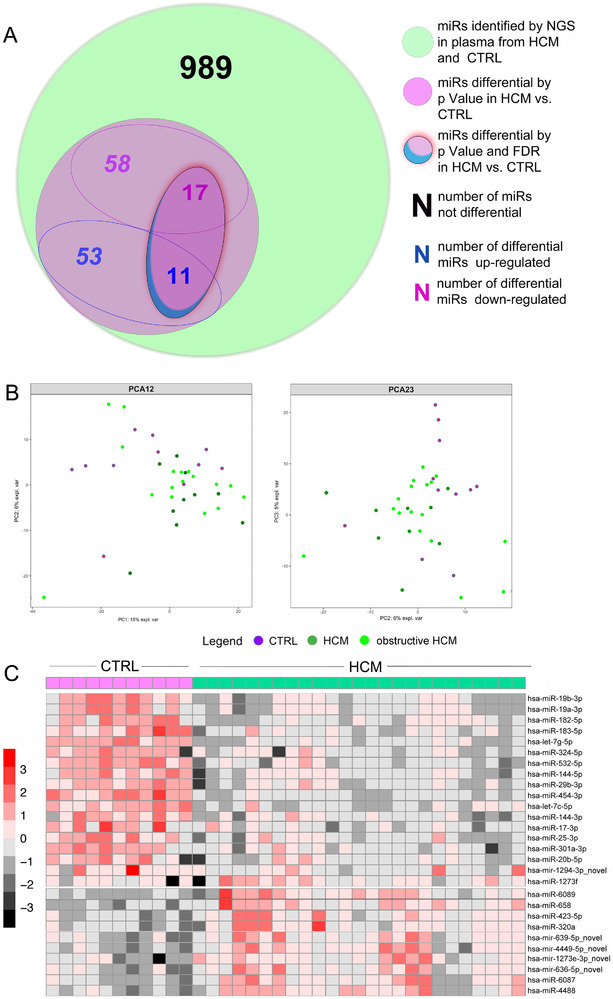
NGS analysis of plasma miRs. Venn diagram representation of the plasma miRs up‐/down ‐regulated in HCM patients vs. CTRL by NGS (A) and corresponding scatterplots of principal component analysis (PCA) of patients and controls screened by NGS (B) showing no remarkable discrete clustering of the non‐obstructive HCM (HCM in the panel) vs. obstructive HCM vs. CTRL. Heatmap of the relative expression of miRs differential by NGS displays the existence of finest difference in miR expressions between HCM and CTRL (C)

To validate NGS performance and exclude false DEmiRs due to high specificity but moderate sensitivity of the assay,^4^ 37 miRs (16 DEmiRs, 21 identified plasma miRs involved in cardiovascular diseases/proposed as biomarkers for cardiomyopathy or heart failure [HF] by other studies) were determined in 22 HCM, 10 CTRL by quantitative real‐time polymerase chain reaction (RT‐qPCR). Eight plasma DEmiRs were confirmed by RT‐qPCR: hsa‐miR‐19a‐3p, hsa‐miR‐20b ‐5p, hsa‐miR‐29b‐3p, hsa‐miR‐126‐5p, hsa‐miR‐144‐3p, hsa‐miR‐454‐3p and hsa‐miR‐4732‐5p were up‐regulated, and hsa‐miR‐182‐5p was down‐regulated (Figure 2A, Table S5). All, except the hsa‐miR‐454‐3p demonstrated acceptable accuracy by receiver operating characteristic (ROC) analysis (Figure 2B). Conversely, six miRs were undetected in all plasma samples (i.e., hsa‐miR‐1273a, hsa‐miR‐1273c, hsa‐miR‐1285‐3p, hsa‐miR‐363‐5p, hsa‐miR‐658, and hsa‐miR‐6089) and 23 miRs showed a comparable expression in HCM and CTRL (Table S5, Figure S3). Our approach showed an overall agreement but incomplete overlapping of NGS and RT‐qPCR results, highlighting the challenge of an accurate quantification.^4^

**FIGURE 2:**
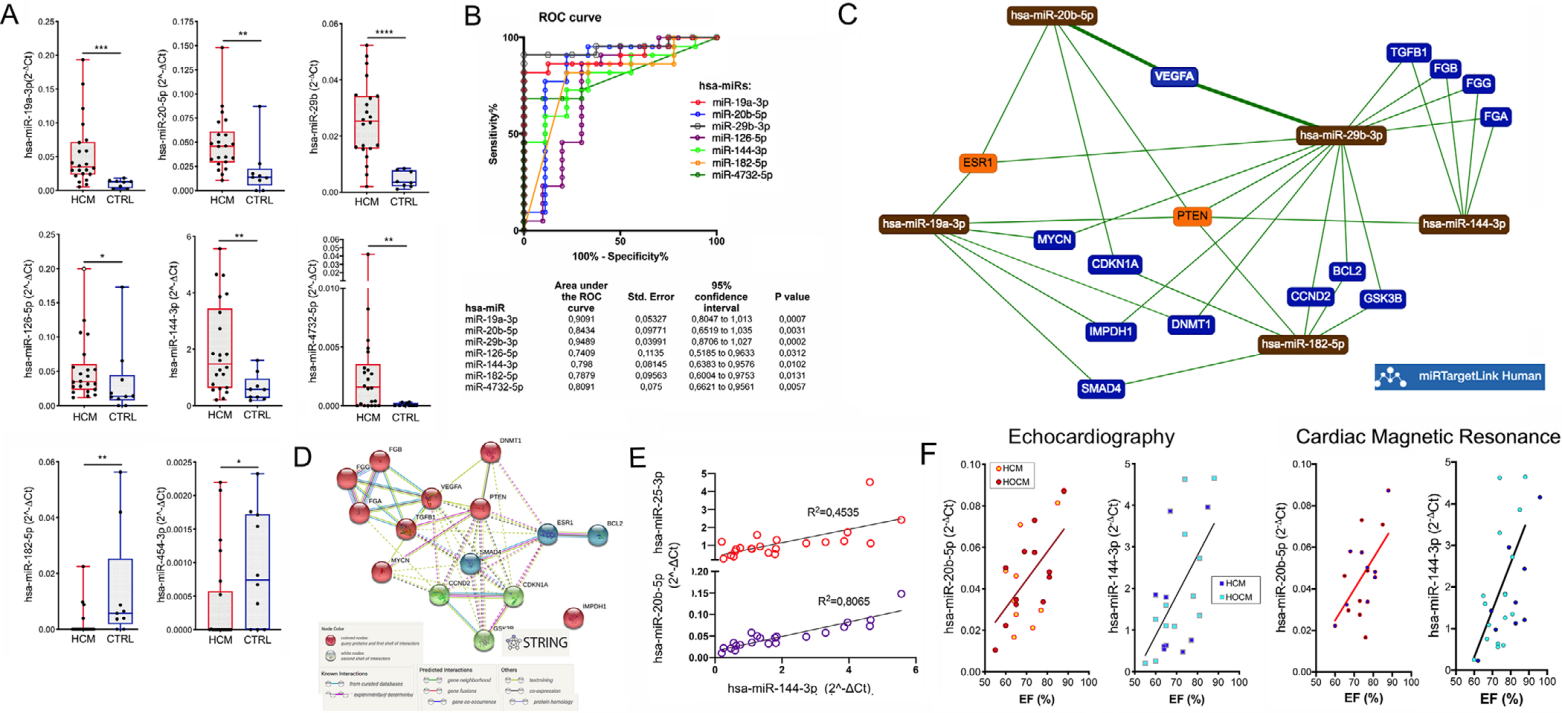
Analysis of plasma miRs by RT‐qPCR and *in silico* engines and association between plasma miRs by RT‐qPCR and left ventricle ejection fraction. Differential plasma miR expression levels in HCM vs. CTRL by RT‐qPCR are plotted (A). Values are presented as boxes (min to max) and dots indicate single sample values. Mann‐Whitney test is applied and significant differences are shown as *p < 0.05 and **p < 0.01, ***p < 0.001, ****p < 0.0001. Receiver‐operator characteristic (ROC) curve analysis of plasma DEmiRs is shown (B). AUC > 0.7 with significant *p* values was considered as threshold for good discriminant performance. The network obtained by miR TargetLink Human for strong interactions among DEmiRs and their putative target genes is shown (C). Orange nodes show target genes associated with three or more miRs, blue nodes those shared by less than three miRs, brown nodes indicate miRs. The interactions among the proteins encoded by target genes drawn by STRING v11 are presented (D). Nodes corresponding to clustered proteins are presented in the same color. Linear relations between hsa‐miR‐144‐3p and hsa‐miR‐20b‐5p or hsa‐miR‐25‐3p in the plasma samples of HCM population are shown (E). Linear relation between the hsa‐miR‐144‐3p and hsa‐miR‐20b‐5p expression levels determined by RT‐qPCR and the left ventricle ejection fraction (EF) assessed by routine echocardiography or cardiac magnetic resonance in patients either with non obstructive (HCM) or obstructive (HOCM) hypertrophic cardiomyopathy is shown (F)

The *in silico* analysis associated with validated plasma DEmiRs 15 predicted target genes (Figure 2C), three interacting protein clusters (Figure 2D), and numerous biological processes (Supplemental_Enrichment 1).

The Spearman's rank coefficient calculation differently correlated pairs of miRs highly‐expressed by RT‐qPCR in HCM and CTRL (Figure S4A), and a significant linear relationship relating hsa‐miR‐144‐3p to both hsa‐miR‐20b‐5p and hsa‐miR‐25‐3p in HCM was found (Figure 2E). These three miRs were interconnected by 93 predicted targets, including PTEN and BCL2L11 genes backed‐up with strong evidence (Figure S4B), and leading to network of 17 proteins involved in pathways of cardiac relevance such as the regulation of cardiac muscle cell proliferation and of response to endoplasmic reticulum stress (Figure S4C). Furthermore, the linear association between plasma levels of hsa‐miR‐144‐3p and hsa‐miR‐20b‐5p and % ejection fraction (EF) found in the HCM population (Figure 2F, Table S6) suggested a role for these two miRs in the regulation of myocardial function.

Thereafter, 20 miRs already validated in the plasma (including all up‐regulated DEmiRs) were determined in septal myectomy samples from 21 obstructive HCM of our cohort and nine donor hearts (ctrl). RT‐qPCR showed three downregulated (hsa‐miR‐144‐3p, hsa‐miR‐451a and hsa‐miR‐223‐3p, Figure 3A) and two up‐regulated (hsa‐miR‐374b‐5p and hsa‐miR‐4485‐3p, Figure 3B) tissue DEmiRs.

**FIGURE 3:**
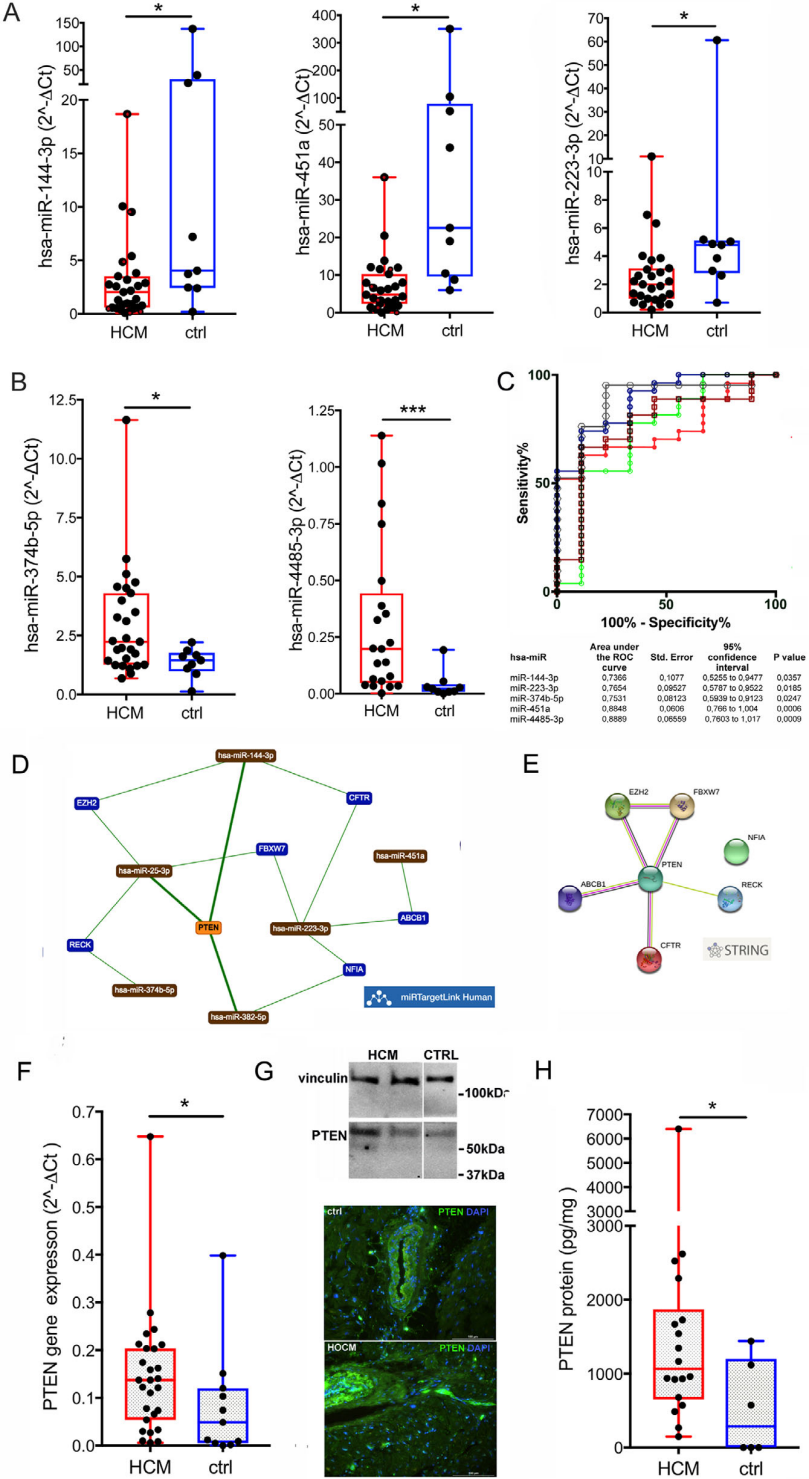
Analysis of myocardial tissue miRs by RT‐qPCR and expression of PTEN. The expression levels of DEmiRs determined by RT‐qPCR in the myocardial tissue samples of HCM vs. ctrl are plotted. The miRs down‐regulated in HCM are in A, those upregulated in B. Receiver‐operator characteristic (ROC) curve analysis of tissue DEmiRs is shown (C). AUC > 0.7 with significant *p* values was considered as threshold for good discriminant performance. The network obtained by miR TargetLink Human for strong interactions among hsa‐miR‐4451, hsa‐miR‐382‐5p, hsa‐miR‐25‐3p, tissue DEmiRs and their putative target genes is presented (D). Orange nodes show target genes associated with three or more miRs, blue nodes those shared by less than three miRs, brown nodes indicate miRs. The corresponding protein network drawn by STRING v11 is shown (E). The upregulation of PTEN gene determined by RT‐qPCR in HCM vs. ctrl tissues is plotted, (F). Representative qualitative evaluation of the protein presence into myocardial tissues from two HCMs and one ctrl by western blot (G, up) is shown. Immunofluorescence images of HCM and ctrl cryosections are displayed (G, down) and PTEN quantification by specific ELISA is plotted (H). Values In A, B, F, and H plots are presented as boxes (min to max), and dots indicate single sample values. Mann‐Whitney test is applied and significant differences are shown as *p < 0.05

The miR144/451 family was associated with extracellular matrix remodeling,^5^ negative regulation of hypertrophy and autophagy,^6^ and cardiac diseases by other reports (e.g., the HUNT study^7^). Our data in HCM showed that hsa‐miR‐144‐3p and hsa‐miR‐451a were linearly related, while hsa‐miR‐4732‐5p undetermined in the majority of samples (Figure S5), confirming a published report in HCM patients,^6^ but also suggesting an opposite trend in plasma and tissue for hsa‐miR‐144‐3p expression, due to increased cardiac release/decreased synthesis. This hints to a role in cardiac remodeling in HCM, deserving further investigations.

Moreover, ROC analysis demonstrated the good performance of 4 out of 5 tissue DEmiRs (Figure 3C).

The other miRs tested in tissues showed comparable expression levels in HCM and ctrl (Figure S6), but four of them were related to HCM clinical phenotypes (Figure S7). Specifically, hsa‐miR‐4451 was linearly associated with interventricular septum thickness, both hsa‐miR‐382‐5p and hsa‐miR‐25‐3p to glomerular filtration rate, and hsa‐miR‐382‐5p negatively and non‐linearly to Troponin T.

The calculation of Spearman correlation between miR pairs showed some positive relationships in HCM tissue (>0.60) with significant linear fits (Figure S8A‐SAC).

I*n silico* analysis of myocardial DEmiRs and of miRs associated with clinical parameters predicted networks of targets genes and interacting proteins (Figures 3D 3E, and S8D) partially superimposable to those drawn for validated plasma DEmiRs, and showed PTEN as a shared target in both HCM plasma and myocardial tissue. Notably, a significant up‐regulation of PTEN gene expression was determined in myectomies from HCM vs. ctrl (*p* = 0.035, Figure 3F) and increased expressed protein amount (Figures 3G and 3H, *p* = 0.047) was also found in HCM samples. PTEN deletion in mice drove variable *in vivo* and *in vitro* effects on cardiomyocyte hypertrophy,^8^ and a pro‐hypertrophic signaling pathway involving miR‐20b and PTEN was proposed in conditions of pressure‐overload cardiac hypertrophy.^8^, ^9^, ^10^ To the best of our knowledge PTEN‐related mechanisms in human HCM have not been elucidated. Based on our results we hypothesize a mechanistic role for DEmiRs in the modulation of PTEN in HCM, possibly in relation to metabolic alterations, and suggesting the need for dedicated mechanistic studies.

In summary (Figure 4), starting from the whole plasma miR transcriptome of HCM and CTRL, we determined the relative and absolute abundance of specific miRs and delineated a preliminary panel of plasma/tissue DEmiRs which included hsa‐miR‐182‐5p, hsa‐miR‐126‐5p, hsa‐miR‐19a‐3p, hsa‐miR‐20b‐5p, hsa‐miR‐29b‐3p, hsa‐miR‐144‐3p, hsa‐miR‐223‐3p, and hsa‐miR‐4485‐3p as potential biomarkers setting HCM apart from other diseases (Table 1 for comparison with other human studies). The DEmiR target genes network drawn by in silico analyses hinted to a role for PTEN in HCM pathogenesis.

**FIGURE 4:**
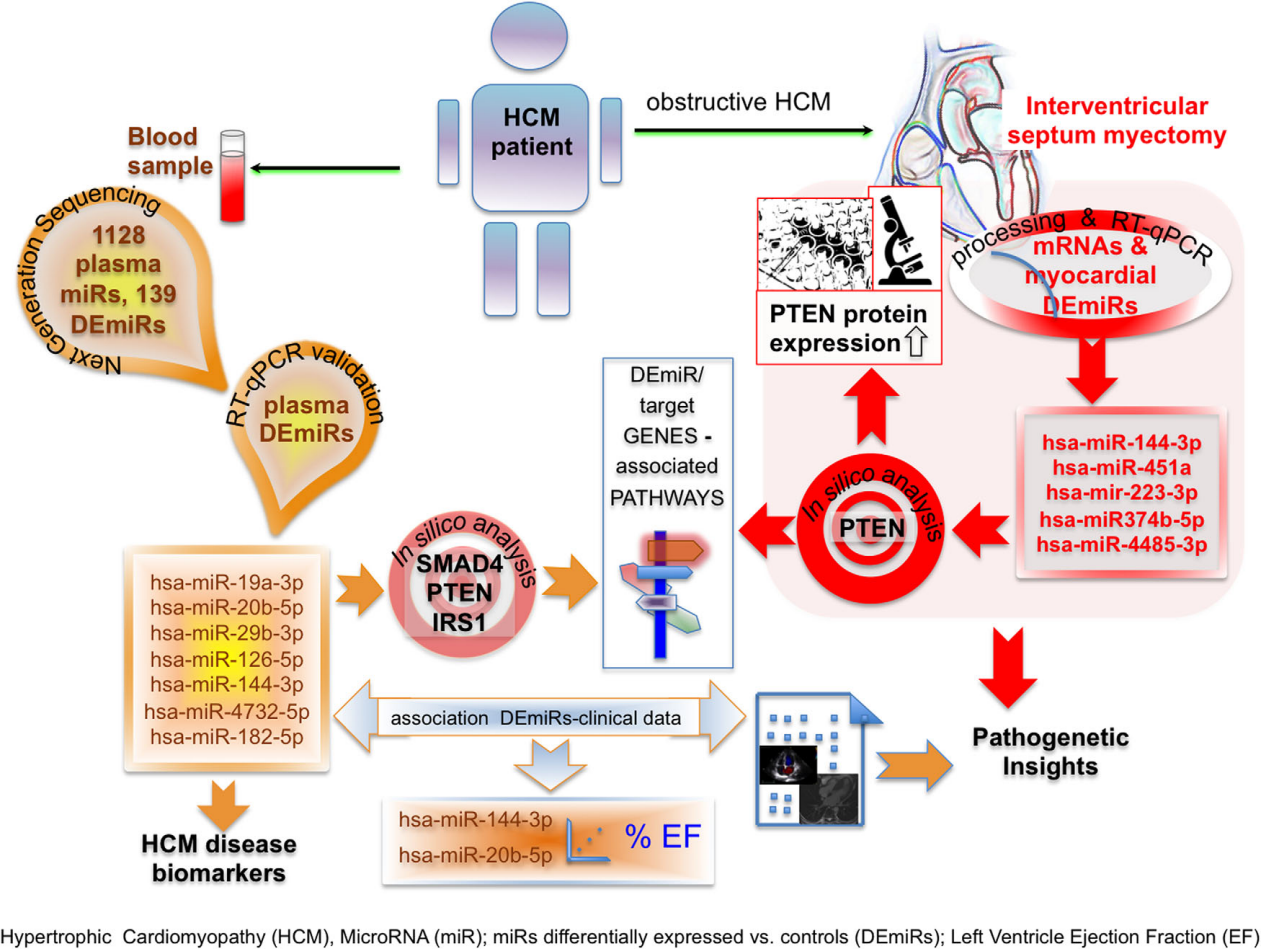
Schematic representation of the study results

**TABLE 1:** DEmiRs: Comparison with published studies

	Plasma						Myocardial tissue			
	HCM (our study)	CAD	HF	AF	AMI	other pathologies	HCM (our study)	other pathologies	References_DOI	References_full citation
miR‐19a	UP				UP	UP (chronic Chagas disease)	NDE	UP (chronic Chagas disease)	https://doi.org/10.3390/ijms20164064	Nonaka, C.K.V. et al, Circulating miRNAs as Potential Biomarkers Associated with Cardiac Remodeling and Fibrosis in Chagas Disease Cardiomyopathy. International journal of molecular sciences, 20.
									https://doi.org/10.31557/apjcp.2020.21.4.975	Mansouri, F. and Seyed Mohammadzad, M.H. Molecular miR‐19a in Acute Myocardial Infarction: Novel Potential Indicators of Prognosis and Early Diagnosis. Asian Pacific journal of cancer prevention: APJCP, 21, 975–982.
									https://doi.org/10.3390/ijms151120355	Zhong, J. et al, Circulating microRNA‐19a as a potential novel biomarker for diagnosis of acute myocardial infarction. International journal of molecular sciences, 15, 20355–20364.
									https://doi.org/10.1038/s41467‐019‐09530‐1	Gao, F. et al, Therapeutic role of miR‐19a/19b in cardiac regeneration and protection from myocardial infarction. Nature communications, 10, 1802.
miR‐20b	UP					DOWN (T2DM)	NDE		https://doi.org/10.1038/s41598‐020‐63606‐3	Wander, P.L. et al, Short Report: Circulating microRNAs are associated with incident diabetes over 10 years in Japanese Americans. Scientific reports, 10, 6509.
									https://doi.org/10.1161/circresaha.110.226357	Zampetaki, A. et al, Plasma microRNA profiling reveals loss of endothelial miR‐126 and other microRNAs in type 2 diabetes. Circulation research, 107, 810–817.
									https://doi.org/10.3892/ijmm.2014.1691	Zhou, J.et al, microRNA expression profiling of heart tissue during fetal development. International journal of molecular medicine, 33, 1250–1260.
miR‐29b	UP				UP	UP (chronic Chagas disease)	NDE	UP (chronic Chagas disease, arrythmogenic right ventricle cardiomyopathy)	https://doi.org/10.3390/ijms20164064	Nonaka, C.K.V. et al, Circulating miRNAs as Potential Biomarkers Associated with Cardiac Remodeling and Fibrosis in Chagas Disease Cardiomyopathy. International journal of molecular sciences, 20.
									https://doi.org/10.1111/cpr.12764	Yang, Q. et al, Aberrant expression of miR‐29b‐3p influences heart development and cardiomyocyte proliferation by targeting NOTCH2. Cell proliferation, 53, e12764.
miR‐126	UP	DOWN	UP	UP	UP	DOWN (T2DM, chronic renal disease)	NDE		https://doi.org/10.4314/ahs.v17i2.22	Wang, X. et al, Expression of miR‐126 and its potential function in coronary artery disease. African health sciences, 17, 474–480.
									https://doi.org/10.1159/000447794	Li, H.Y. et al, Plasma MicroRNA‐126‐5p is Associated with the Complexity and Severity of Coronary Artery Disease in Patients with Stable Angina Pectoris. Cellular physiology and biochemistry: international journal of experimental cellular physiology, biochemistry, and pharmacology, 39, 837–846.
									https://doi.org/10.1038/s41598‐019‐41101‐8	Fourdinier, O. et al, Serum levels of miR‐126 and miR‐223 and outcomes in chronic kidney disease patients. Scientific reports, 9, 4477.
miR‐144	UP	UP			UP	UP (arrythmogenic right ventricle cardiomyopathy)	DOWN	UP (arrythmogenic right ventricle cardiomyopathy)	https://doi.org/10.1016/j.rec.2017.05.013	de Gonzalo‐Calvo, D. et al, Epigenetic Biomarkers and Cardiovascular Disease: Circulating MicroRNAs. Revista espanola de cardiologia (English ed.), 70, 763–769.
									https://doi.org/10.1016/j.yjmcc.2016.05.009	Bye, A. et al, Circulating microRNAs predict future fatal myocardial infarction in healthy individuals ‐ The HUNT study. Journal of molecular and cellular cardiology, 97, 162–168.
									https://doi.org/10.1371/journal.pone.0226164	Abu‐Halima, M. et al, Micro‐RNA signatures in monozygotic twins discordant for congenital heart defects. PloS one, 14, e0226164.
									https://doi.org/10.1111/jcmm.12380	Song, L. et al, MiR‐451 is decreased in hypertrophic cardiomyopathy and regulates autophagy by targeting TSC1. Journal of cellular and molecular medicine, 18, 2266–2274.
miR‐182	DOWN	UP	UP			UP (arrythmogenic right ventricle cardiomyopathy)	NDE	DOWN (arrythmogenic right ventricle cardiomyopathy)	https://doi.org/10.1042/cs20100043	Taurino, C. et al, Gene expression profiling in whole blood of patients with coronary artery disease. Clinical science (London, England : 1979), 119, 335–343.
									https://doi.org/10.2459/jcm.0000000000000233	Cakmak, H.A. et al, The prognostic value of circulating microRNAs in heart failure: preliminary results from a genome‐wide expression study. Journal of cardiovascular medicine (Hagerstown, Md.), 16, 431–437.
									https://doi.org/10.1038/srep21228	Li, N. et al, miR‐182 Modulates Myocardial Hypertrophic Response Induced by Angiogenesis in Heart. Scientific reports, 6, 21228.
miR‐223	NDE						DOWN		https://doi.org/10.1155/2015/592512	Barsanti, C. et al, Differential regulation of microRNAs in end‐stage failing hearts is associated with left ventricular assist device unloading. BioMed research international, 2015, 592512.
miR‐223 (Cont.)									https://doi.org/10.1155/2015/943659	Chuang, T.Y. et al, MicroRNA‐223 Expression is Upregulated in Insulin Resistant Human Adipose Tissue. Journal of diabetes research, 2015, 943659.
									https://doi.org/10.1093/cvr/cvq010	Lu, H., Buchan, R.J. and Cook, S.A., MicroRNA‐223 regulates Glut4 expression and cardiomyocyte glucose metabolism. Cardiovascular research, 86, 410–420.
miR‐374b	NDE		DOWN		UP (STEMI vs. NSTEMI)		UP	DOWN (calcific aortic stenosis: valves)	https://doi.org/10.4172/2327‐4972.1000108	Ward, J.A. et al, Circulating Cell and Plasma microRNA Profiles Differ between Non‐ST‐Segment and ST‐Segment‐Elevation Myocardial Infarction. Family medicine & medical science research, 2, 108.
									https://doi.org/10.1007/s11010‐017‐2947‐7	Xu, H.X. et al, Differential Expression of MicroRNAs in Calcific Aortic Stenosis. Clinical laboratory, 63, 1163–1170.
miR‐451a	NDE				NDE		DOWN		https://doi.org/10.1111/jcmm.12380	Song, L. et al, MiR‐451 is decreased in hypertrophic cardiomyopathy and regulates autophagy by targeting TSC1. Journal of cellular and molecular medicine, 18, 2266–2274.
miR‐454	DOWN					UP (DCM in children) ***	NDE		https://doi.org/10.3109/1354750x.2015.1118533	Enes Coşkun, M. et al, Plasma microRNA profiling of children with idiopathic dilated cardiomyopathy. Biomarkers: biochemical indicators of exposure, response, and susceptibility to chemicals, 21, 56–61.
miR‐4485	NDE						UP		/	/
miR‐4732	UP					UP (congenital heart defetcs)	NA		https://doi.org/10.1371/journal.pone.0226164	Abu‐Halima, M .et al, Micro‐RNA signatures in monozygotic twins discordant for congenital heart defects. PloS one, 14, e0226164.

LEGEND: NDE, not differentially expressed vs. ctrl; UP, up‐regulated; DOWN, down‐regulated; NA, undetermined by RT‐qPCR.

## AUTHOR CONTRIBUTIONS

Conceptualization: C.F. and P.G.C.; Data curation: D.L., G.B, I.O. and O.R.; Formal analysis: M.R. and C.F.; Investigation: M.L., D.L., G.B., G.d'A., G.B, F.deC. and C.F.; Methodology: M.L., D.L.; Project administration: C.F.; Supervision: P.G.C.; Visualization,: M.L., M.R. and C.F.; Writing original draft: M.L., D.L., C.F. and P.G.C.; Writing review & editing: I.O., C.F. and P.G.C. All authors have read and agreed to the published version of the manuscript.

## Supporting information

Supporting Information

Supporting Information

Supporting Information
