# Supplementary material for: Plasmatic and myocardial microRNA profiles in patients with Hypertrophic Cardiomyopathy
Source: Clin Transl Med. 2021 Jul 19;11(7):e435. doi: 10.1002/ctm2.435 (PMC8287979; doi:10.1002/ctm2.435)

Supplementary Materials to

**Plasmatic and myocardial miRs in patients with Hypertrophic Cardiomyopathy**

Lombardi Maria, Lazzeroni Davide, Benedetti Giulia, Bertoli Gloria, Lazarevic Dejan Riba Michela, DeCobelli Francesco, Rimoldi Ornella, d'Amati Giulia, Olivotto Iacopo, Foglieni Chiara and Camici Paolo

**Materials and Methods**

***Population***

Patients with both non-obstructive and obstructive HCM, defined according to existing guidelines^1^, were prospectively screened and characterized at San Raffaele Hospital, Milan and at Careggi Hospital, Florence. Thirty-six patients were enrolled according to the inclusion and exclusion criteria listed in Supplemental Table 1 and, blood samples obtained from 25 of them. Anonymized blood samples from 11 healthy volunteers matched for age and gender served as controls (CTRL).

In HCM patients, baseline demographic, clinical and routine evaluations (ECG, echocardiography), and cardiac magnetic resonance (CMR) were performed at baseline (flowchart in Supplemental Figure1; summary of clinical and imaging data in Supplemental Table 2).

Of the 36, 27 patients with obstructive HCM and advanced heart failure symptoms (NYHA Class ≥ III), syncope or pre-syncope due to LV obstruction, exercise hypotension, drug-refractory symptoms, or presence of moderate-to-severe systolic anterior motion-related mitral regurgitation were scheduled for surgical myectomy in Milan and Florence. Fourteen obstructive patients underwent both plasma and tissue screening. The septal myectomy samples were harvested and immediately processed. Control tissue samples (ctrl) were provided by 9 donor hearts discarded from transplantation because of non-cardiac technical reasons, collected at Policlinico Umberto I Hospital, Rome. According to the Italian law, no data about the donors was available. The control hearts were extensively sampled for paraffin inclusion. Hematoxylin-Eosin -stained sections were examined under light microscope, to exclude the presence of cardiomyocyte hypertrophy, necrosis or degenerative changes, significant fibrosis or inflammatory infiltrates ^2^.

The study protocol conformed to the ethical guidelines of the 1975 Declaration of Helsinki and was approved by the institutional ethics committees. All patients signed an informed consent.

***Cardiac magnetic resonance*** ***imaging***

Cardiac magnetic resonance imaging (CMR) was performed in HCM patients without history of cancer, allergy to gadolinium, claustrophobia, previous known left ventricular (LV) ejection fraction <35%, inability to lay flat for at least 30 minutes A 1.5-T whole-body scanner (Achieva; Philips Medical System, Best, NL) and a 5-elements cardiac phased-array coil with respiratory and ECG gating (SENSE Cardiac, Philips Medical Systems) were used. Late gadolinium enhancement (LGE) after Gadolinium injection was performed. The myocardial wall thickness and diameters, recoding maximum wall thickness, septal thickness and posterior wall thickness were manually measured. Left atrial volume (LAV) was measured using the biplane area-length method ^3^ and normalized for body surface area (LAV/BSA). Semi-automatic software (ViewForum release 4.2, Philips Medical System calculated end-diastolic and end-systolic LV volume, ejection fraction, and LV mass. Volumes and mass were also normalized for body surface area.

***Plasma samples***

Blood samples (10mL) were collected in BD Vacutainer® EDTA tubes, processed under sterile conditions to obtain plasma within 15min from collection, and aliquots (200μL/aliquot) stored at -80°C.

***Tissue processing***

Myectomies were processed within 2h from ablation and tissues cut into 2mm thick slices perpendicularly to the endocardium. Part of them was included in paraffin for diagnostic purposes, part snap‑frozen in liquid nitrogen. Serial cryosections (10 or 20μm thick) were obtained by Leica CM1850 cryostat (Leica Microsystems GmbH, Wetzlar, Germany), and collected either onto slides to be submitted to immunofluorescence, or in vials to be processed for obtaining RNA and protein extracts.

***RNA isolation from plasma and tissues***

Plasma *miR*-enriched fraction was isolated with miRNeasy serum/plasma miR isolation kit (Qiagen, Hilden, Germany). Spectrophotometric measurements excluded the presence of contaminating hemolysis (i.e. absence of relevant peaks at 414nm and 576nm wavelength ^4^) were performed at Infinite F200 microplate reader, (TECAN Group Ltd, Männedorf, Switzerland).

Total RNA, including miR-enriched fraction and mRNA was extracted from cryosections using miRNeasy mini kit and RNeasy mini kit (Qiagen, Hilden, Germany), respectively. Total RNA was quantified at NanoDrop™ 1000 (Thermo Fisher Scientific, Inc., Waltham, MA, US).

Synthesis of cDNA was carried out either by TaqMan® Advanced miRNA Assay (plasma and tissue miRs) or High Capacity RNA-to-cDNA Kit (tissue mRNA) (all from Invitrogen, Carlsbad, CA, USA).

***Plasma miRs sequencing and in silico analysis***

Next-generation sequencing (NGS) ^5^ of plasma miR profile was performed with Illumina Sequencer, and mature miR sequences were aligned using SMARTer smRNA-Seq technology (Clontech/Takara Bio Inc, Shiga, Japan) and run in single read 75 nt, aligned using sRNAbench ^6, 7^. Three sequencing runs were performed to mimic a “real life situation”, where samples will not be contemporary available. In each run, at least 3 samples were repeated for allowing batch correction during the statistical analysis. The full miRnome expression profiles were obtained and the levels of miRs expressed in at least 10 samples were analyzed to identify differentially expressed miRs in HCM vs. CTRL. Multivariate, exploratory analysis by Principal Component Analysis (PCA) and clustering was performed.

*In silico* analysis was performed on differential miRs using *miRTargetLink Human* ^8^ for identifying effective microRNA target sites, and *STRING* v11 ^9^ for encoded protein pathway networking.

***Quantitative reverse transcription Polymerase Chain Reaction on plasma and tissues***

Technical validation of selected plasma miRs was performed comparing relative levels of miRs on de novo extracted plasma aliquots from the same HCM and CTRL individuals evaluated by NGS. The determined miRs were: 16 DEmiRs significant for both p Value and FDR by NGS (all those with potentially shared interactions by in silico analysis and two - hsa-miR-658 and hsa-miR-6089 - with no predicted interaction, Supplemental Table 4); 14 miRs randomly chosen among those with significant p Value but not significant FDR by NGS and reported by other studies to be involved in cardiovascular disease pathogenesis ^10-12^ (Supplemental Table 4) and 7 miRs (hsa-miR-1-3p, hsa-miR-382-5p, miR-409-3p, hsa-miR-432-5p, hsa-miR-451a, hsa-miR-4451 and hsa-miR-4485-3p) proposed as biomarkers for cardiomyopathy or HF by other studies ^13-17^.

The putative DEmiRs by NGS with no commercially available probe were excluded from the validation as well those that failed to show any shared interaction by the in silico analysis with *miRTargetLink Human* *^8^*(Supplemental table 4).

Twenty miRs were analyzed also in myectomy tissues from 21 patients enrolled in Milan (see Population paragraph). Specifically, from the list of miRs validated by RT-qPCR into plasma we determined in the tissue 7 DEmiRs, the hsa-miR-19b-3p and hsa-miR-451a (belonging to the same family of hsa-miR-19a-3p and hsa-miR-144-3p, respectively), the hsa-miR-25-3p (linearly related to hsa-miR-144-3p into plasma), and 10 randomly -selected miRs. Six tissue samples from HCM patients enrolled in Florence served for blind validation.

The quantitative reverse transcription Polymerase Chain Reaction (RT-qPCR) was carried out by TaqMan® Fast Advanced Master Mix with Taqman primer/Fam-labeled probes (Applied Biosystems, Foster City, CA, USA) (Supplemental Table 3). Samples were run in triplicate, and miR levels were normalized to that of hsa-miR16-5p.and hsa-miR-103a-3p for plasma and tissue, respectively ^18-20^. Reproducibility of results was tested by repeating the RT-qPCR determination for 5 DEmiRs randomly selected among those identified by NGS.

Gene expression of PTEN was evaluated in tissue samples by RT-qPCR using TaqMan® Universal Master Mix II with Taqman primer/Fam-labeled probes (Hs02621230_s1). Gene levels were normalized to that of βactin (Hs01060665_g1). Relative expression was determined using the ΔCt method.

All the procedures were carried out according to the manufacturers’ instructions.

***Western blot***

Tissue was lysed in Cell Lysis buffer supplemented with phenylmethylsulfonyl fluoride (Cell Signaling Technology, Danvers, MA, US) and the protein concentration determined by Bradford method. After denaturation in sample buffer containing NuPAGE™ Sample Reducing Agent (5min, 95°C), the protein extracts (15μg) were loaded and resolved 4-15% SDS-PAGE, then transferred onto 0.2μm nitrocellulose membrane. After blocking of non-specific binding in 5% milk buffer (1h, RT) the membranes were incubated with rabbit-anti- and rabbit-anti-vinculin (both diluted 1:1000, 4°C, overnight, clone D4.3, and clone E1E9V, respectively, Cell signaling Technology),, followed by goat-anti-rabbit-HRP-conjugated and Clarity™ western ECL substrate. Band optical density of PTEN was detected using UVITEC Image system (Cleaver Scientific) and normalized vs. vinculin.

***Enzyme-Linked Immunosorbent* *Assay***

Human PTEN content was quantified in myocardial tissues by simpleStep ELISA kits (ab206979, Abcam, [Cambridge, UK](https://www.google.com/search?rls=com.microsoft:it-IT:IE-SearchBox&q=Cambridge&stick=H4sIAAAAAAAAAOPgE-LSz9U3ME0ySC43VOIAsS3NLYq0tLKTrfTzi9IT8zKrEksy8_NQOFYZqYkphaWJRSWpRcWLWDmdE3OTijJT0lN3sDICALVAxwFTAAAA&sa=X&ved=2ahUKEwiEoq6u0M_rAhXFnVwKHTjkA1IQmxMoATAbegQIEhAD)) following manufacturers’ instructions. Luminescence was measured on Infinite F200 microplate reader (TECAN Group Ltd, Männedorf, Switzerland). Samples were run in triplicate.

***Immunofluorescence***

Cryosections were fixed in acetone for 10min RT, permeabilized with 0.1% tritonX-100 in phosphate buffer solution (10min, RT), and non-specific binding was blocked by 1% bovine serum albumin (30min, RT). Sections were incubated with rabbit-anti-human PTEN (1:100, 4°C, overnight, GeneTex, Irvine, CA, US) revealed by donkey anti-rabbit-IgG (H+L) AlexaFluor 488 (45min, RT, Invitrogen, Carlsbad, CA, US). Nuclei were stained with 4',6-diamidino-2-phenylindole (DAPI, 0.2 nmol/L, 10min, RT). Fluorsave-mounted sections were analyzed under Eclipse 55i microscope equipped with a DS-L1 camera (Nikon, Tokyo, Japan). Single-channel acquired images were merged by AdobePhotoshopCS.

**Statistical analysis**

Modeling of the miRs count dataset and differential expression evaluation was done using edgeR ^21^, applying a correction to reduce batch effects in NGS data.

The normality of RT-qPCR and clinical data distribution was assessed by Shapiro-Wilk normality test. Correlation among clinical and experimental variables was analyzed by Spearman test, simple linear regression or nonlinear fit test. Data from HCM and control samples were compared by Mann Whitney unpaired t-test or Kruskal-Wallis with Dunn's Multiple Comparison post-hoc test. The value of RT-qPCR for validating DEmiRs was assessed by calculating the area under the receiver-operator characteristic (ROC) curve (AUC; 0.7 <AUC<0.8 accuracy of the test acceptable, AUC; 0.8 ≤AUC<0.9 excellent, AUC≥0.9 outstanding) ^22^. Probability values <0.05 were considered significant. The Prism 8.2 software was used in all analyses.

Limitation

The assessment of miR modules associated with different disease phenotypes is beyond the scope of this study. Evaluation of the miR levels in relation to disease stage could be object of a dedicated project in a large HCM population.

**References**

1. Elliott PM, Anastasakis A, Borger MA, et al. 2014 ESC Guidelines on diagnosis and management of hypertrophic cardiomyopathy: the Task Force for the Diagnosis and Management of Hypertrophic Cardiomyopathy of the European Society of Cardiology (ESC). *European heart journal*. Oct 14 2014;35(39):2733-79. doi:10.1093/eurheartj/ehu284

2. Lombardi M, Lazzeroni D, Pisano A, et al. Mitochondrial Energetics and Ca2(+)-Activated ATPase in Obstructive Hypertrophic Cardiomyopathy. *Journal of clinical medicine*. Jun 9 2020;9(6)doi:10.3390/jcm9061799

3. Lang RM, Bierig M, Devereux RB, et al. Recommendations for chamber quantification: a report from the American Society of Echocardiography's Guidelines and Standards Committee and the Chamber Quantification Writing Group, developed in conjunction with the European Association of Echocardiography, a branch of the European Society of Cardiology. *Journal of the American Society of Echocardiography : official publication of the American Society of Echocardiography*. Dec 2005;18(12):1440-63. doi:10.1016/j.echo.2005.10.005

4. Kirschner MB, Kao SC, Edelman JJ, et al. Haemolysis during sample preparation alters microRNA content of plasma. *PloS one*. 2011;6(9):e24145. doi:10.1371/journal.pone.0024145

5. Koppers-Lalic D. *sRNAbench: profiling of small RNAs and its sequence variants in single or multi-species high-throughput experiments*. vol 1. 2014.

6. Aparicio-Puerta E, Lebrón R, Rueda A, et al. sRNAbench and sRNAtoolbox 2019: intuitive fast small RNA profiling and differential expression. *Nucleic acids research*. Jul 2 2019;47(W1):W530-w535. doi:10.1093/nar/gkz415

7. Coenen-Stass AML, Magen I, Brooks T, et al. Evaluation of methodologies for microRNA biomarker detection by next generation sequencing. *RNA Biology*. 2018/08/03 2018;15(8):1133-1145. doi:10.1080/15476286.2018.1514236

8. Hamberg M, Backes C, Fehlmann T, et al. MiRTargetLink--miRNAs, Genes and Interaction Networks. *International journal of molecular sciences*. Apr 14 2016;17(4):564. doi:10.3390/ijms17040564

9. Szklarczyk D, Gable AL, Lyon D, et al. STRING v11: protein-protein association networks with increased coverage, supporting functional discovery in genome-wide experimental datasets. *Nucleic acids research*. Jan 8 2019;47(D1):D607-d613. doi:10.1093/nar/gky1131

10. McManus DD, Lin H, Tanriverdi K, et al. Relations between circulating microRNAs and atrial fibrillation: data from the Framingham Offspring Study. *Heart rhythm*. Apr 2014;11(4):663-9. doi:10.1016/j.hrthm.2014.01.018

11. Shah R, Tanriverdi K, Levy D, et al. Discordant Expression of Circulating microRNA from Cellular and Extracellular Sources. *PloS one*. 2016;11(4):e0153691. doi:10.1371/journal.pone.0153691

12. Wang K, Long B, Liu F, et al. A circular RNA protects the heart from pathological hypertrophy and heart failure by targeting miR-223. *European heart journal*. Sep 1 2016;37(33):2602-11. doi:10.1093/eurheartj/ehv713

13. Chen MC, Chang TH, Chang JP, et al. Circulating miR-148b-3p and miR-409-3p as biomarkers for heart failure in patients with mitral regurgitation. *International journal of cardiology*. Nov 1 2016;222:148-154. doi:10.1016/j.ijcard.2016.07.179

14. Huang YM, Li WW, Wu J, Han M, Li BH. The diagnostic value of circulating microRNAs in heart failure. *Experimental and therapeutic medicine*. Mar 2019;17(3):1985-2003. doi:10.3892/etm.2019.7177

15. Scrimgeour NR, Wrobel A, Pinho MJ, Høydal MA. microRNA-451a prevents activation of matrix metalloproteinases 2 and 9 in human cardiomyocytes during pathological stress stimulation. *American journal of physiology Cell physiology*. Jan 1 2020;318(1):C94-c102. doi:10.1152/ajpcell.00204.2019

16. Song L, Su M, Wang S, et al. MiR-451 is decreased in hypertrophic cardiomyopathy and regulates autophagy by targeting TSC1. *Journal of cellular and molecular medicine*. Nov 2014;18(11):2266-74. doi:10.1111/jcmm.12380

17. Zeng Z, Wang K, Li Y, et al. Down-regulation of microRNA-451a facilitates the activation and proliferation of CD4(+) T cells by targeting Myc in patients with dilated cardiomyopathy. *The Journal of biological chemistry*. Apr 7 2017;292(14):6004-6013. doi:10.1074/jbc.M116.765107

18. Donati S, Ciuffi S, Brandi ML. Human Circulating miRNAs Real-time qRT-PCR-based Analysis: An Overview of Endogenous Reference Genes Used for Data Normalization. *International journal of molecular sciences*. Sep 5 2019;20(18)doi:10.3390/ijms20184353

19. Sucharov CC, Kao DP, Port JD, et al. Myocardial microRNAs associated with reverse remodeling in human heart failure. *JCI insight*. Jan 26 2017;2(2):e89169. doi:10.1172/jci.insight.89169

20. Wang X, Zhang X, Yuan J, et al. Evaluation of the performance of serum miRNAs as normalizers in microRNA studies focused on cardiovascular disease. *Journal of thoracic disease*. May 2018;10(5):2599-2607. doi:10.21037/jtd.2018.04.128

21. Robinson MD, McCarthy DJ, Smyth GK. edgeR: a Bioconductor package for differential expression analysis of digital gene expression data. *Bioinformatics (Oxford, England)*. Jan 1 2010;26(1):139-40. doi:10.1093/bioinformatics/btp616

22. Mandrekar JN. Receiver operating characteristic curve in diagnostic test assessment. *Journal of thoracic oncology : official publication of the International Association for the Study of Lung Cancer*. Sep 2010;5(9):1315-6. doi:10.1097/JTO.0b013e3181ec173d

**Supplemental Table1 Patients' selection criteria**

| Inclusioni criteria | Exclusion criteria |
| --- | --- |
| Diagnosis of HCM:   - maximum LV wall thickness superior or equal to 1.5cm by Echocardiography - age >18 years and <80 years - symptomatic patients, in hemodynamic stability | - CAD, AMI - other causes of potential CMD - incessant ventricular arrhythmias - prior septal myectomy and/or alcohol ablation - overt LV systolic dysfunction with LV EF < 50% - uncontrolled arterial hypertension - BMI >32 kg/m2 - Severe renal impairment (GFR <29 mL/min; Creatinine level >2.5mg/dL; BUN >60mg/dL) - IDDM - moderate/severe hepatic impairment / insufficiency - females of childbearing potential not using adequate contraceptive precautions |

**Legend:** HCM hypertrophic cardiomyopathy; LV, left ventricle; CAD, coronary artery disease; AMI acute myocardial infarction; CMD, coronary microvascular dysfunction; BMI, body mass index; EF, ejection fraction; GFR, glomerular filtration rate; BUN, blood urea nitrogen; IDDM, insulin-dependent diabetes mellitus

**Supplemental Table 2 Data of patients with HCM**

|  | **HCM**  **(n=36)** |
| --- | --- |
| **Demographic and clinical data** |  |
| Age (years): M (SD) | 59 (11) |
| Gender: % male | 61 |
| BMI (Kg/m^2^), M (SD) | 27 (5) |
| NYHA ≥ II: % | 75 |
| **Medical Therapy** |  |
| Beta-blockers: % | 86 |
| ACE-i: % | 16 |
| CCB: % | 11 |
| Diuretics: % | 53 |
| **Echocardiography** |  |
| IVS thickness (mm): M (SD) | 20 (5) |
| LV-EDV (ml): M (SD) | 104 (54) |
| LV-EF (%): M (SD) | 68 (8) |
| E/A ratio: M (SD) | 0.97 (0.7) |
| E/e’ ratio: M (SD) | 9 (6) |
| Moderate-to-severe mitral regurgitation: % | 53 |
| SAM-related LVOT-max gradient at rest (mmHg): M (SD) | 73 (33) |
|  |  |
| **Cardiac Magnetic Resonance** | **HCM**  **(n=18)** |
| Left ventricle |  |
| IVS thickness (mm): M (SD) | 23 (6) |
| LV-EDVi (ml/m2): M (SD) | 73 (21) |
| LV-ESVi (ml/m2): M (SD) | 20 (10) |
| LV-EF (%): M (SD) | 74(7) |
| EDWMi (gr) | 119 (58) |
| Right ventricle |  |
| RV-EDVi (ml/m2): M (SD) | 55 (13) |
| RV-ESVi (ml/m2): M (SD) | 19 (5) |
| RV-EF (%): M (SD) | 66 (7) |

**Legend**: M, mean; SD, standard deviation; %, percentage; HCM hypertrophic cardiomyopathy; BMI, body mass index; NYHA, New York Heart Association class; ACE-i, angiotensin-converting-enzyme system inhibitors; CCB, calcium channel blockers; IVS, interventricular septum; LV, left ventricular; EDV, end diastolic volume; EF, ejection fraction; SAM, Systolic anterior motion; Max, maximum; LVOT, left ventricular outflow tract; EDVi, end diastolic volume indexed; ESVi, end systolic volume indexed; EDWMi; end-diastolic-wall-mass indexed; RV, right ventricle.

| **Supplemental Table 3 Probes for miR determination by RT-qPCR** | | |
| --- | --- | --- |
| **Assay ID** | **Assay name** |  |
| [478575_mir](https://www.thermofisher.com/order/genome-database/details/microrna/478575_mir?CID=&ICID=&subtype=) | hsa-let-7a-5p |  |
| [478577_mir](https://www.thermofisher.com/order/genome-database/details/microrna/478577_mir?CID=&ICID=&subtype=) | hsa-let-7c-5p |  |
| [478578_mir](https://www.thermofisher.com/order/genome-database/details/microrna/478578_mir?CID=&ICID=&subtype=) | hsa-let-7f-5p |  |
| 478580_mir | hsa-let-7g-5p |  |
| [477888_mir](https://www.thermofisher.com/order/genome-database/details/microrna/477888_mir?CID=&ICID=&subtype=) | hsa-miR-126-5p |  |
| [478676_mir](https://www.thermofisher.com/order/genome-database/details/microrna/478676_mir?CID=&ICID=&subtype=) | hsa-miR-1273a |  |
| [478677_mir](https://www.thermofisher.com/order/genome-database/details/microrna/478677_mir?CID=&ICID=&subtype=) | hsa-miR-1273c |  |
| [478687_mir](https://www.thermofisher.com/order/genome-database/details/microrna/478687_mir?CID=&ICID=&subtype=) | hsa-miR-1285-3p |  |
| [477820_mir](https://www.thermofisher.com/order/genome-database/details/microrna/477820_mir?CID=&ICID=&subtype=) | hsa-miR-1-3p |  |
| [477913_mir](https://www.thermofisher.com/order/genome-database/details/microrna/477913_mir?CID=&ICID=&subtype=) | hsa-miR-144-3p |  |
| [477919_mir](https://www.thermofisher.com/order/genome-database/details/microrna/477919_mir?CID=&ICID=&subtype=) | hsa-miR-151a-3p |  |
| 477935_mir | hsa-miR-182-5p |  |
| [477937_mir](https://www.thermofisher.com/order/genome-database/details/microrna/477937_mir?CID=&ICID=&subtype=) | hsa-miR-183-5p |  |
| [479228_mir](https://www.thermofisher.com/order/genome-database/details/microrna/479228_mir?CID=&ICID=&subtype=) | hsa-miR-19a-3p |  |
| [478264_mir](https://www.thermofisher.com/order/genome-database/details/microrna/478264_mir?CID=&ICID=&subtype=) | hsa-miR-19b-3p |  |
| [477804_mir](https://www.thermofisher.com/order/genome-database/details/microrna/478253_mir?CID=&ICID=&subtype=) | hsa-miR-20b-5p |  |
| [477983_mir](https://www.thermofisher.com/order/genome-database/details/microrna/477983_mir?CID=&ICID=&subtype=) | hsa-miR-223-3p |  |
| [477994_mir](https://www.thermofisher.com/order/genome-database/details/microrna/477994_mir?CID=&ICID=&subtype=) | hsa-miR-25-3p |  |
| [478369_mir](https://www.thermofisher.com/order/genome-database/details/microrna/478369_mir?CID=&ICID=&subtype=) | hsa-miR-29b-3p |  |
| 477815_mir | hsa-miR-301a-3p |  |
| [478594_mir](https://www.thermofisher.com/order/genome-database/details/microrna/478594_mir?CID=&ICID=&subtype=) | hsa-miR-320a |  |
| [478840_mir](https://www.thermofisher.com/order/genome-database/details/microrna/478840_mir?CID=&ICID=&subtype=) | hsa-miR-363-5p |  |
| [478389_mir](https://www.thermofisher.com/order/genome-database/details/microrna/478389_mir?CID=&ICID=&subtype=) | hsa-miR-374b-5p |  |
| [478078_mir](https://www.thermofisher.com/order/genome-database/details/microrna/478078_mir?CID=&ICID=&subtype=) | hsa-miR-382-5p |  |
| [478084_mir](https://www.thermofisher.com/order/genome-database/details/microrna/478084_mir?CID=&ICID=&subtype=) | hsa-miR-409-3p |  |
| [478090_mir](https://www.thermofisher.com/order/genome-database/details/microrna/478090_mir?CID=&ICID=&subtype=) | hsa-miR-423-5p |  |
| [478101_mir](https://www.thermofisher.com/order/genome-database/details/microrna/478101_mir?CID=&ICID=&subtype=) | hsa-miR-432-5p |  |
| [480822_mir](https://www.thermofisher.com/order/genome-database/details/microrna/480822_mir?CID=&ICID=&subtype=) | hsa-miR-4451 |  |
| [479430_mir](https://www.thermofisher.com/order/genome-database/details/microrna/479430_mir?CID=&ICID=&subtype=) | hsa-miR-4485-3p |  |
| 478107_mir | hsa-miR-451a |  |
| [478329_mir](https://www.thermofisher.com/order/genome-database/details/microrna/478329_mir?CID=&ICID=&subtype=) | hsa-miR-454-3p |  |
| [478119_mir](https://www.thermofisher.com/order/genome-database/details/microrna/478119_mir?CID=&ICID=&subtype=) | hsa-miR-4732-5p |  |
| [478966_mir](https://www.thermofisher.com/order/genome-database/details/microrna/478966_mir?CID=&ICID=&subtype=) | hsa-miR-5096 |  |
| [480184_mir](https://www.thermofisher.com/order/genome-database/details/microrna/480184_mir?CID=&ICID=&subtype=) | hsa-miR-6089 |  |
| [479140_mir](https://www.thermofisher.com/order/genome-database/details/microrna/479140_mir?CID=&ICID=&subtype=) | hsa-miR-658 |  |
| [479164_mir](https://www.thermofisher.com/order/genome-database/details/microrna/479164_mir?CID=&ICID=&subtype=) | hsa-miR-718 |  |
| [478590_mir](https://www.thermofisher.com/order/genome-database/details/microrna/478590_mir?CID=&ICID=&subtype=) | hsa-miR-98-5p |  |
| [478253_mir](https://www.thermofisher.com/order/genome-database/details/microrna/478253_mir?CID=&ICID=&subtype=) | hsa-miR-103a-3p |  |

| **Supplemental Table 4** miRs differentially expressed in plasma samples from HCM and CTRL | | | | | | | | | |  |
| --- | --- | --- | --- | --- | --- | --- | --- | --- | --- | --- |
| **DEmiRs** | **NGS results** | | | | | **In silico analysis**  number of  **shared interactions** | |  |  |  |
|  | **log Fold Change** | **log CPM** | **P Value** | **FDR** | **weak + strong** | | **strong** | |  |  |
| hsa-miR-19b-3p | -2,6344 | 14,450 | <0,00001 | 0,00153 | 591 | | 19 | |  |  |
| hsa-miR-19a-3p | -2,6377 | 13,128 | <0,00001 | 0,00170 | 536 | | 15 | |  |  |
| hsa-miR-182-5p | -4,8530 | 5,560 | <0,00001 | 0,00170 | 79 | | 12 | |  |  |
| hsa-miR-183-5p | -3,1474 | 8,472 | 0,00002 | 0,00344 | 184 | | 7 | |  |  |
| hsa-let-7g-5p | -2,1591 | 13,231 | 0,00002 | 0,00344 | 292 | | 9 | |  |  |
| hsa-miR-324-5p | -2,4874 | 8,446 | 0,00002 | 0,00344 | 107 | | 0 * | |  |  |
| hsa-miR-532-5p | -2,5431 | 8,745 | 0,00002 | 0,00344 | 25 | | 0 * | |  |  |
| hsa-miR-144-5p | -2,4288 | 10,045 | 0,00003 | 0,00434 | 15 | | 0 * | |  |  |
| hsa-miR-6089 | 2,3827 | 10,237 | 0,00004 | 0,00448 | 60 | | 0 * | |  |  |
| hsa-mir-639-5p_novel | 2,2578 | 9,487 | 0,00004 | 0,00459 | NA * | | NA * | |  |  |
| hsa-miR-29b-3p | -2,1162 | 10,788 | 0,00019 | 0,01896 | 93 | | 19 | |  |  |
| hsa-miR-454-3p | -3,0127 | 8,755 | 0,00020 | 0,01896 | 353 | | 1 | |  |  |
| hsa-let-7c-5p | -2,154 | 11,968 | 0,00024 | 0,02051 | 362 | | 9 | |  |  |
| hsa-miR-144-3p | -2,0997 | 10,669 | 0,00041 | 0,03336 | 105 | | 7 | |  |  |
| hsa-miR-17-3p | -3,5280 | 5,543 | 0,00057 | 0,04279 | 74 | | 0 * | |  |  |
| hsa-mir-4449-5p_novel | 1,6933 | 13,807 | 0,00066 | 0,04299 | NA * | | NA * | |  |  |
| hsa-miR-658 | 3,2789 | 5,720 | 0,00069 | 0,04299 | 64 | | 0 * | |  |  |
| hsa-miR-423-5p | 1,6634 | 15,201 | 0,00073 | 0,04299 | 141 | | 0 * | |  |  |
| hsa-miR-320a | 1,8396 | 10,634 | 0,00073 | 0,04299 | 239 | | 5 | |  |  |
| hsa-miR-1273f | 2,2364 | 8,199 | 0,00084 | 0,04299 | 109 | | 0 * | |  |  |
| hsa-miR-25-3p | -1,5270 | 15,376 | 0,00087 | 0,04299 | 194 | | 8 | |  |  |
| hsa-miR-301a-3p | -2,8203 | 8,032 | 0,00089 | 0,04299 | 353 | | 3 | |  |  |
| hsa-miR-6087 | 1,6560 | 11,339 | 0,00097 | 0,04299 | 91 | | 0 * | |  |  |
| hsa-mir-1273e-3p_novel | 2,5137 | 10,644 | 0,00097 | 0,04299 | NA * | | NA * | |  |  |
| hsa-mir-636-5p_novel | 1,6446 | 11,822 | 0,00099 | 0,04299 | NA * | | NA * | |  |  |
| hsa-mir-1294-3p_novel | -5,285 | 4,254 | 0,00105 | 0,04351 | NA * | | NA * | |  |  |
| hsa-miR-4488 | 1,8223 | 13,246 | 0,00108 | 0,04351 | 38 | | 0 * | |  |  |
| hsa-miR-20b-5p | -1,9491 | 9,311 | 0,00119 | 0,04637 | 413 | | 7 | |  |  |
|  | | | | | | | | | | |

**LEGEND** DEmiRs, differentially expressed miRs; HCM, hypertrophic cardiomyopathy; NGS, next generation sequencing; CPM, count per millions; FDR false discovery rate; NA: not assessed = no data on miRTargetLink Human website; *: no post-NGS validation

| **Supplemental Table 5 miRs differential in HCM vs. CTRL (p value significant, FDR not significant)** **by NGS.** The miRs successively validated in RT-qPCR are labeled in green | | | | |
| --- | --- | --- | --- | --- |
| **miRs** | **log Fold Change** | **log CPM** | **p** | **FDR** |
| hsa-miR-17-5p | -1,695742374 | 11,321 | 0,00198 | 0,07244 |
| hsa-miR-1290 | 1,879200052 | 16,242 | 0,00199 | 0,07244 |
| hsa-miR-98-5p | -2,109538368 | 9,513 | 0,00209 | 0,07377 |
| hsa-miR-1285-3p | 2,522020554 | 9,502 | 0,00223 | 0,07377 |
| hsa-miR-6126 | 1,560249075 | 13,412 | 0,00233 | 0,07377 |
| hsa-miR-93-5p | -1,547248475 | 12,720 | 0,00235 | 0,07377 |
| hsa-miR-374b-5p | -2,187329959 | 9,827 | 0,00244 | 0,07377 |
| hsa-let-7f-5p | -2,018506427 | 13,475 | 0,00250 | 0,07377 |
| hsa-miR-1246 | 1,801707367 | 16,610 | 0,00254 | 0,07377 |
| hsa-miR-4750-5p | 4,552938447 | 4,229 | 0,00255 | 0,07377 |
| hsa-miR-30e-3p | -1,929691599 | 9,251 | 0,00269 | 0,07585 |
| hsa-miR-483-5p | 1,910101023 | 8,599 | 0,00291 | 0,08015 |
| hsa-miR-4532 | 1,468551659 | 15,839 | 0,00386 | 0,10055 |
| hsa-miR-3195-3p_novel | 1,609928612 | 10,753 | 0,00398 | 0,10055 |
| hsa-miR-4800-5p | 1,878570864 | 10,129 | 0,00399 | 0,10055 |
| hsa-miR-3960 | 1,671422666 | 9,775 | 0,00401 | 0,10055 |
| hsa-mir-196a-1-3p_novel | -4,277700737 | 4,590 | 0,00424 | 0,10402 |
| hsa-miR-1538 | 3,763874371 | 4,295 | 0,00435 | 0,10457 |
| hsa-let-7a-5p | -1,938991546 | 14,881 | 0,00475 | 0,11165 |
| hsa-miR-877-5p | 1,499453214 | 7,420 | 0,00502 | 0,11555 |
| hsa-miR-16-2-3p | -1,442151583 | 9,874 | 0,00564 | 0,12683 |
| hsa-miR-223-3p | -1,934425878 | 13,989 | 0,00573 | 0,12683 |
| hsa-miR-26b-5p | -1,667497043 | 9,375 | 0,00603 | 0,13102 |
| hsa-miR-126-5p | -1,675391955 | 14,645 | 0,00623 | 0,13263 |
| hsa-miR-18b-5p | -2,393456032 | 6,257 | 0,00677 | 0,13830 |
| hsa-miR-142-5p | -1,472492884 | 12,757 | 0,00693 | 0,13830 |
| hsa-miR-374a-3p | -2,148375872 | 6,213 | 0,00707 | 0,13830 |
| hsa-miR-106a-5p | -1,614828938 | 8,791 | 0,00719 | 0,13830 |
| hsa-mir-4533-3p_novel | 3,022483691 | 4,896 | 0,00721 | 0,13830 |
| hsa-miR-576-5p | -1,554875716 | 7,870 | 0,00728 | 0,13830 |
| hsa-miR-378a-5p | -2,228454305 | 6,068 | 0,00735 | 0,13830 |
| hsa-mir-4767-3p_novel | 1,738856521 | 6,790 | 0,00754 | 0,13953 |
| hsa-miR-1273c | 2,167871735 | 7,927 | 0,00772 | 0,14051 |
| hsa-miR-3665 | 1,824546773 | 6,005 | 0,00821 | 0,14706 |
| hsa-miR-337-5p | 3,451196724 | 4,507 | 0,00868 | 0,15152 |
| hsa-miR-101-3p | -1,812562319 | 9,896 | 0,00872 | 0,15152 |
| hsa-let-7b-5p | -1,441539314 | 15,089 | 0,00908 | 0,15537 |
| hsa-miR-151a-3p | -1,791288133 | 11,254 | 0,00938 | 0,15807 |
| hsa-miR-548d-3p | -2,841923965 | 5,188 | 0,00975 | 0,16194 |
| hsa-mir-1234-5p_novel | 1,379342173 | 10,092 | 0,01029 | 0,16823 |
| hsa-miR-106b-3p | -1,370061288 | 8,152 | 0,01046 | 0,16823 |
| hsa-miR-5100 | 1,650749642 | 8,636 | 0,01058 | 0,16823 |
| hsa-miR-1273a | 2,158857729 | 12,242 | 0,01143 | 0,17921 |
| hsa-miR-4787-5p | 1,377905496 | 7,961 | 0,01164 | 0,18005 |
| hsa-let-7c-3p | -3,339331023 | 4,088 | 0,01231 | 0,18670 |
| hsa-miR-3120-3p | 3,371388664 | 4,559 | 0,01240 | 0,18670 |
| hsa-miR-320b | 1,425008445 | 8,886 | 0,01257 | 0,18674 |
| hsa-miR-363-5p | 2,575830025 | 4,878 | 0,01330 | 0,19504 |
| hsa-miR-4732-5p | 1,356650961 | 8,544 | 0,01463 | 0,20627 |
| hsa-miR-6881-3p | -3,388867028 | 4,267 | 0,01487 | 0,20627 |
| hsa-mir-3159-3p_novel | 1,94252059 | 5,369 | 0,01492 | 0,20627 |
| hsa-miR-3613-5p | -1,718075673 | 7,151 | 0,01501 | 0,20627 |
| hsa-miR-5096 | 1,904439904 | 6,397 | 0,01522 | 0,20627 |
| hsa-miR-374a-5p | -1,799731519 | 8,092 | 0,01532 | 0,20627 |
| hsa-miR-185-5p | -1,217007594 | 11,468 | 0,01535 | 0,20627 |
| hsa-miR-194-5p | -1,409781359 | 8,903 | 0,01657 | 0,21925 |
| hsa-miR-15b-3p | -1,28458176 | 10,229 | 0,01705 | 0,21925 |
| hsa-miR-1255b-5p | -1,678576115 | 6,258 | 0,01721 | 0,21925 |
| hsa-miR-320c | 1,749509279 | 6,573 | 0,01726 | 0,21925 |
| hsa-miR-192-5p | -1,377650136 | 9,448 | 0,01736 | 0,21925 |
| hsa-miR-3178 | 1,525838562 | 6,228 | 0,01763 | 0,21925 |
| hsa-mir-8059-3p_novel | 3,323819042 | 4,263 | 0,01771 | 0,21925 |
| hsa-miR-6505-3p | -3,406532689 | 4,193 | 0,01787 | 0,21925 |
| hsa-miR-3925-5p | 2,922962244 | 4,985 | 0,01823 | 0,22136 |
| hsa-miR-1273g-3p | 1,368596876 | 8,901 | 0,01891 | 0,22442 |
| hsa-miR-143-3p | -1,29005009 | 8,597 | 0,01896 | 0,22442 |
| hsa-miR-93-3p | -1,27620608 | 7,679 | 0,01908 | 0,22442 |
| hsa-miR-101-5p | -3,549470153 | 4,397 | 0,02004 | 0,23323 |
| hsa-miR-20a-5p | -1,508774854 | 8,837 | 0,02059 | 0,23719 |
| hsa-miR-3916 | 1,863232886 | 6,543 | 0,02093 | 0,23867 |
| hsa-miR-3940-5p | 2,781860204 | 4,749 | 0,02153 | 0,24306 |
| hsa-miR-4742-3p | -2,940000167 | 4,492 | 0,02201 | 0,24604 |
| hsa-miR-7-1-3p | -1,258726188 | 8,442 | 0,02237 | 0,24763 |
| hsa-miR-18a-5p | -1,885487024 | 7,463 | 0,02267 | 0,24850 |
| hsa-miR-3667-5p | 3,53819915 | 4,576 | 0,02340 | 0,25397 |
| hsa-miR-6511a-3p | -1,429256091 | 7,290 | 0,02397 | 0,25778 |
| hsa-miR-4429 | 2,312087466 | 5,536 | 0,02484 | 0,26390 |
| hsa-miR-6761-5p | 2,377722479 | 5,275 | 0,02501 | 0,26390 |
| hsa-miR-5589-3p | -3,248286859 | 4,179 | 0,02535 | 0,26502 |
| hsa-let-7i-5p | -1,112046488 | 11,665 | 0,02689 | 0,27856 |
| hsa-mir-5095-3p_novel | 1,849485657 | 5,047 | 0,02744 | 0,28167 |
| hsa-mir-1972-1-5p_novel | 2,48466786 | 5,040 | 0,02865 | 0,28878 |
| hsa-mir-1972-2-5p_novel | 2,48466786 | 5,040 | 0,02865 | 0,28878 |
| hsa-mir-4512-5p_novel | 1,610467154 | 8,874 | 0,02991 | 0,29886 |
| hsa-miR-548ac | -2,813300106 | 4,114 | 0,03099 | 0,30694 |
| hsa-miR-6877-5p | 2,564966068 | 4,754 | 0,03190 | 0,31121 |
| hsa-miR-3141 | 1,542598783 | 5,539 | 0,03198 | 0,31121 |
| hsa-miR-4651 | 2,202906069 | 5,065 | 0,03277 | 0,31618 |
| hsa-miR-5189-5p | 2,414004878 | 4,796 | 0,03308 | 0,31647 |
| hsa-miR-6515-5p | -3,123181797 | 4,305 | 0,03586 | 0,33949 |
| hsa-miR-4753-5p | -2,618497132 | 4,501 | 0,03608 | 0,33949 |
| hsa-miR-7-5p | -1,175493123 | 8,367 | 0,03661 | 0,34096 |
| hsa-miR-5698 | 2,880900404 | 4,413 | 0,03699 | 0,34096 |
| hsa-miR-3197 | 1,529009213 | 7,202 | 0,03719 | 0,34096 |
| hsa-miR-4492 | 1,273209274 | 7,754 | 0,03746 | 0,34096 |
| hsa-miR-429 | -2,78778572 | 4,755 | 0,03775 | 0,34096 |
| hsa-miR-3200-3p | -2,346405271 | 5,047 | 0,03816 | 0,34149 |
| hsa-miR-6779-5p | 2,891721988 | 4,253 | 0,03849 | 0,34149 |
| hsa-miR-4449 | -2,081761392 | 5,937 | 0,03872 | 0,34149 |
| hsa-mir-618-3p_novel | -2,68263779 | 4,227 | 0,04042 | 0,35128 |
| hsa-miR-190a-5p | -2,031543849 | 6,083 | 0,04045 | 0,35128 |
| hsa-miR-103a-2-5p | -1,936136142 | 5,175 | 0,04126 | 0,35559 |
| hsa-miR-6075 | 2,68073355 | 4,244 | 0,04235 | 0,36223 |
| hsa-miR-210-3p | -1,198331266 | 6,443 | 0,04360 | 0,37014 |
| hsa-miR-20a-3p | -2,125154847 | 5,575 | 0,04414 | 0,37107 |
| hsa-miR-186-5p | -1,2730597 | 9,951 | 0,04437 | 0,37107 |
| hsa-miR-6891-5p | 2,37796976 | 4,903 | 0,04515 | 0,37483 |
| hsa-miR-6780b-5p | 2,78112307 | 4,376 | 0,04677 | 0,38544 |
| hsa-mir-548n-3p_novel | -2,589909983 | 4,127 | 0,04825 | 0,39473 |
| hsa-miR-642b-3p | 2,491929408 | 4,881 | 0,04908 | 0,39688 |
| hsa-miR-194-3p | -3,167437061 | 4,639 | 0,04921 | 0,39688 |

| **Supplemental Table 6** Statistical comparison between plasma miRs in HCM, and CTRL samples by RT-qPCR   \|  \| **HCM vs. CTRL** \| \| \| \| --- \| --- \| --- \| --- \| \| **miR** \| **p Value §** \| \| \| \| hsa-miR-1-3p \| 0,204 \| ns \| \| \| hsa-let-7a-5p \| 0,325 \| ns \| \| \| hsa-let-7c-5p \| 0,1209 \| ns \| \| \| hsa-let-7f-5p \| 0,7394 \| ns \| \| \| hsa-let-7g-5p \| 0,2766 \| ns \| \| \| hsa-miR-126-5p \| 0,0305 \| * \| \| \| hsa-miR-144-3p \| 0,009 \| ** \| \| \| hsa-miR-151a-3p \| 0,4047 \| ns \| \| \| hsa-miR-182-5p \| 0,0024 \| ** \| \| \| hsa-miR-183-5p \| 0,2634 \| ns \| \| \| hsa-miR-19a-3p \| 0,0003 \| *** \| \| \| hsa-miR-19b-3p \| 0,254 \| ns \| \| \| hsa-miR-20b-5p \| 0,0021 \| ** \| \| \| hsa-miR-223-3p \| 0,1634 \| ns \| \| \| hsa-miR-25-3p \| 0,3126 \| ns \| \| \| hsa-miR-29b-3p \| <0,0001 \| **** \| \| \| hsa-miR-301a-3p \| 0,6198 \| ns \| \| \| hsa-miR-320a \| 0,3453 \| ns \| \| \| hsa-miR-374b-5p \| 0,9446 \| ns \| \| \| hsa-miR-382-5p \| 0,8711 \| ns \| \| \| hsa-miR-409-3p \| 0,2842 \| ns \| \| \| hsa-miR-423-5p \| 0,0524 \| ns \| \| \| hsa-miR-432-5p \| 0,9893 \| ns \| \| \| hsa-miR-4451 \| 0,1041 \| ns \| \| \| hsa-miR-4485-3p \| 0,503 \| ns \| \| \| hsa-miR-451a \| 0,5929 \| ns \| \| \| hsa-miR-454-3p \| 0,0406 \| * \| \| \| hsa-miR-4732-5p \| 0,0033 \| ** \| \| \| hsa-miR-5096 \| 0,252 \| ns \| \| \| hsa-miR-718 \| 0,269 \| ns \| \| hsa-miR-98-5p \| 0,5482 \| ns \| \|  \|  \|  \|   **§** Kruskal-Wallis test |  |
| --- | --- | --- | --- | --- | --- | --- | --- | --- | --- | --- | --- | --- | --- | --- | --- | --- | --- | --- | --- | --- | --- | --- | --- | --- | --- | --- | --- | --- | --- | --- | --- | --- | --- | --- | --- | --- | --- | --- | --- | --- | --- | --- | --- | --- | --- | --- | --- | --- | --- | --- | --- | --- | --- | --- | --- | --- | --- | --- | --- | --- | --- | --- | --- | --- | --- | --- | --- | --- | --- | --- | --- | --- | --- | --- | --- | --- | --- | --- | --- | --- | --- | --- | --- | --- | --- | --- | --- | --- | --- | --- | --- | --- | --- | --- | --- | --- | --- | --- | --- | --- | --- | --- | --- | --- | --- | --- | --- | --- | --- | --- | --- | --- | --- | --- | --- | --- | --- | --- | --- | --- | --- | --- | --- | --- | --- | --- | --- | --- | --- | --- | --- | --- | --- | --- |

**Supplemental Table 7** Correlation miR levels - Ejection Fraction

|  | Correlation | | Linear Regression | | |
| --- | --- | --- | --- | --- | --- |
| **EF% by Echocardiography in HCM (n=21) vs.** | Spearman R | p | R^2^ | F | p |
| hsa-miR-20b-5p | 0.4884 | 0.0247* | 0.3349 | 9.565 | 0.0060* |
| hsa-miR-144-3p | 0,6144 | 0,003** | 0.3665 | 10.99 | 0.0036 |
|  |  |  |  |  |  |
| **EF% by CMR in HCM (n=17) vs.** |  |  |  |  |  |
| hsa-miR-20b-5p | 0,6003 | 0,0123* | 0.3305 | 7.403 | 0.0158* |
| hsa-miR-144-3p | 0,6950 | 0,0026** | 0.3492 | 8.047 | 0.0125* |
|  |  |  |  |  |  |
| asterisk(s) = p significancy |  |  |  |  |  |


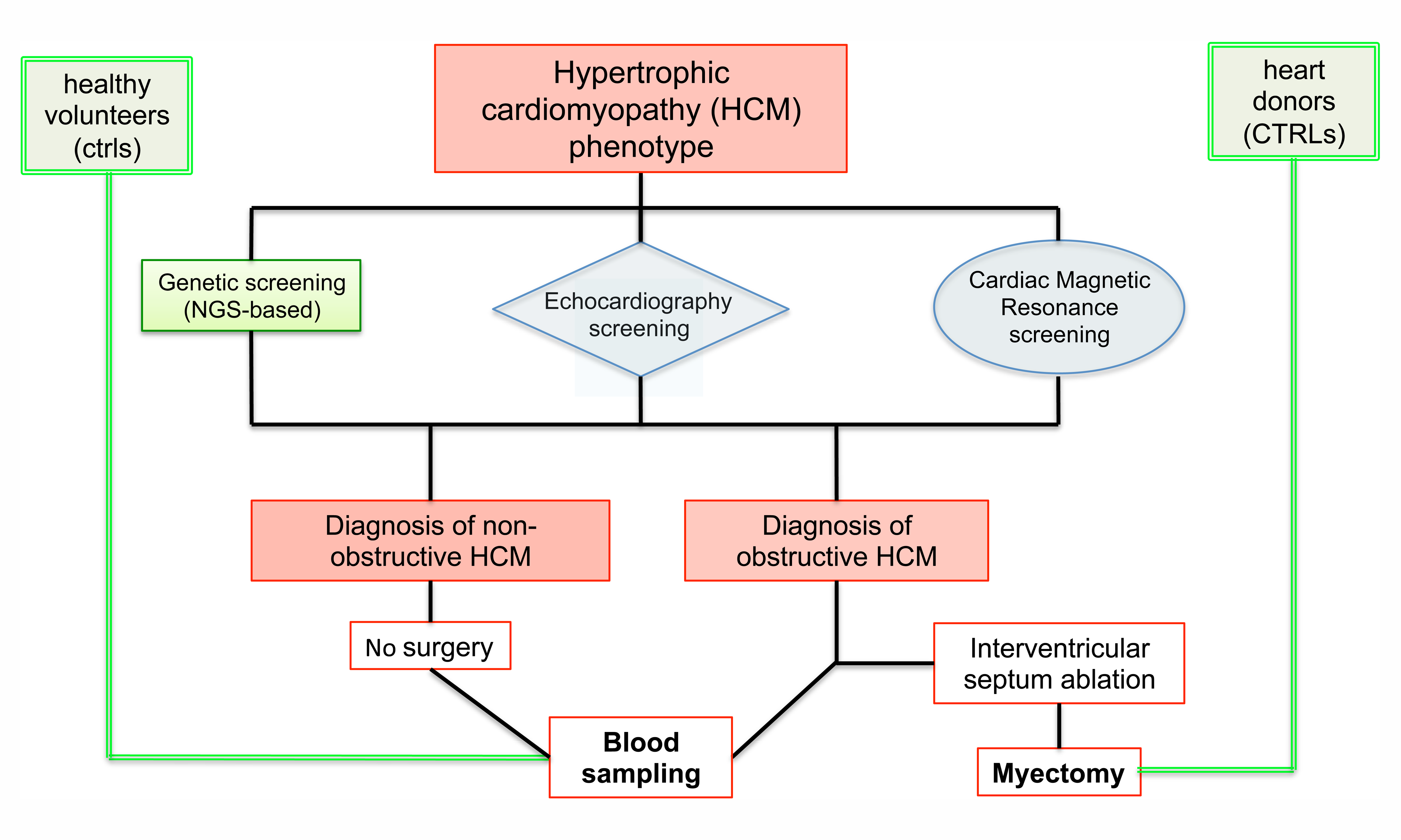


**Supplemental Figure 1 Population**

The characterization and sampling of the studied population is represented as flowchart


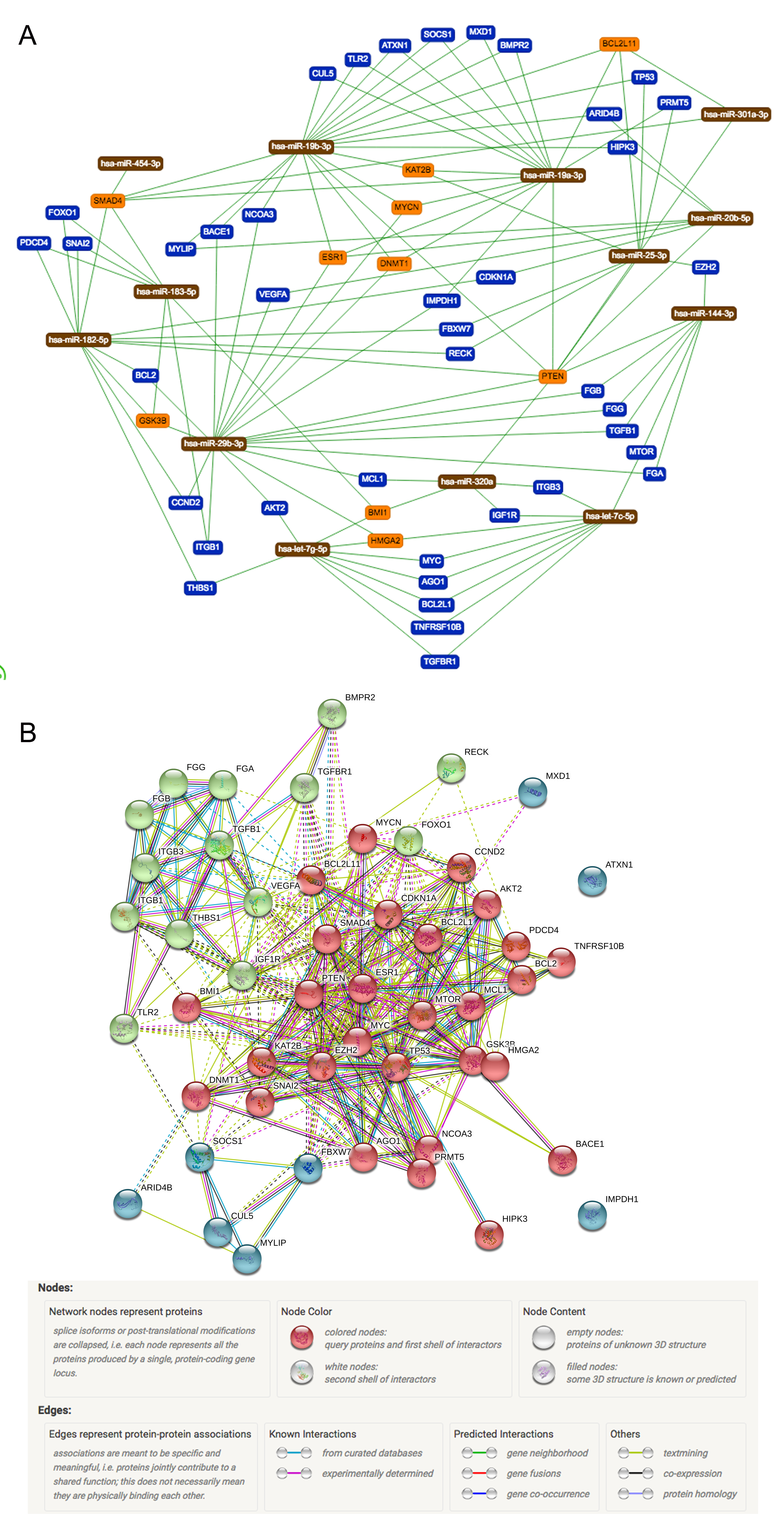


**Supplemental Figure 2:** **In silico analysis of plasma miRs** by **NGS**

The network obtained by miR TargetLink Human for strong interactions, showing connections among the 13 differential miRs and their potential target genes is presented (**A**). Orange nodes show target genes associated to 3 or more miRs, blue nodes those shared by less than 3 miRs, brown nodes indicate miRs. The protein network obtained by STRING v11 from genes showed in **A** is displayed (**B**). Nodes corresponding to clustered proteins are presented in the same color.

**
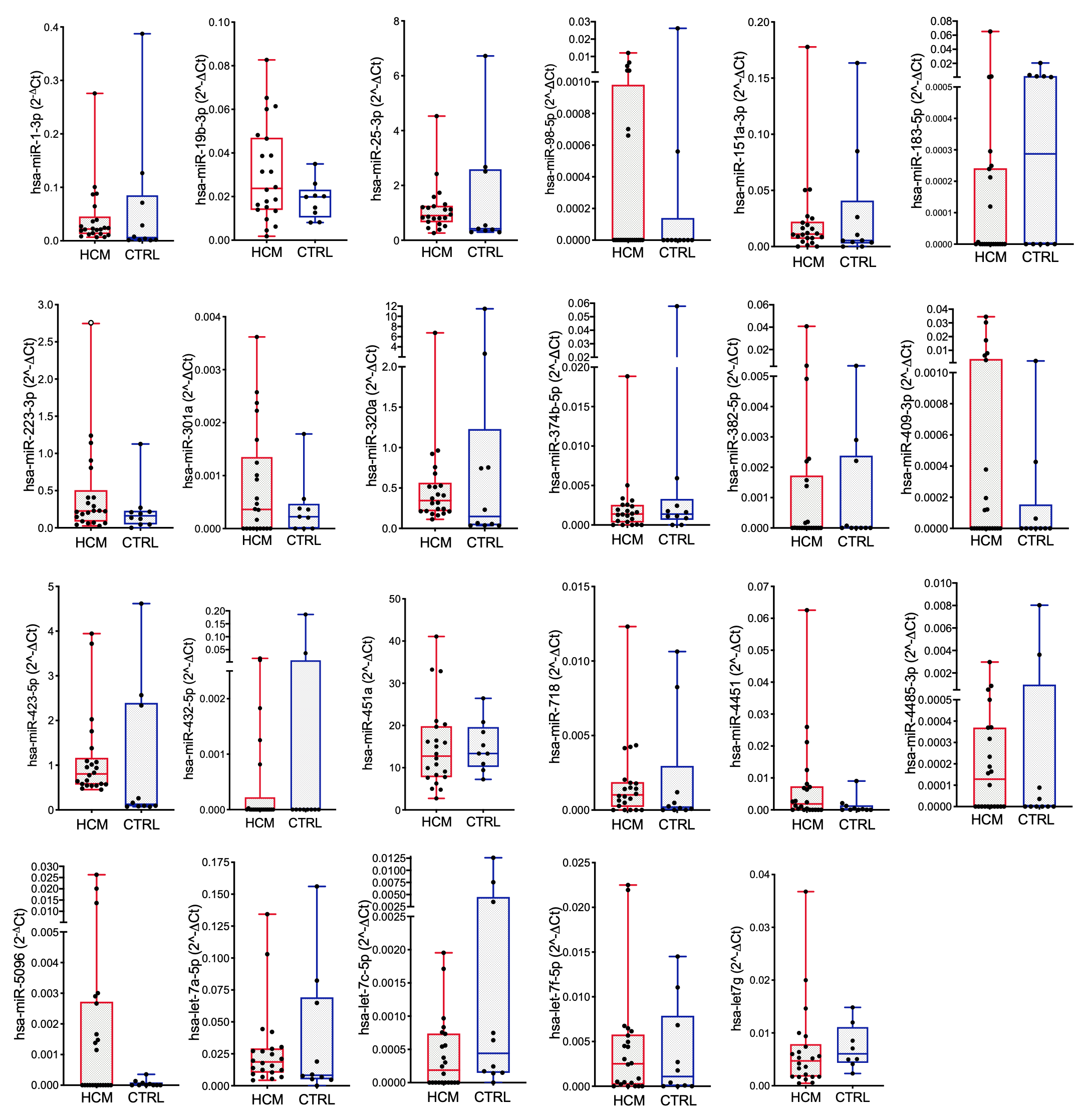
**

**Supplemental Figure 3 Analysis of plasma miRs by RT-qPCR**

Expression levels of plasma DEmiRs by NGS that are not diffremtially expressed by RT-qPCR in HCM vs. CTRL are shown. Values are presented as boxes (min to max) and dots indicate single sample values.


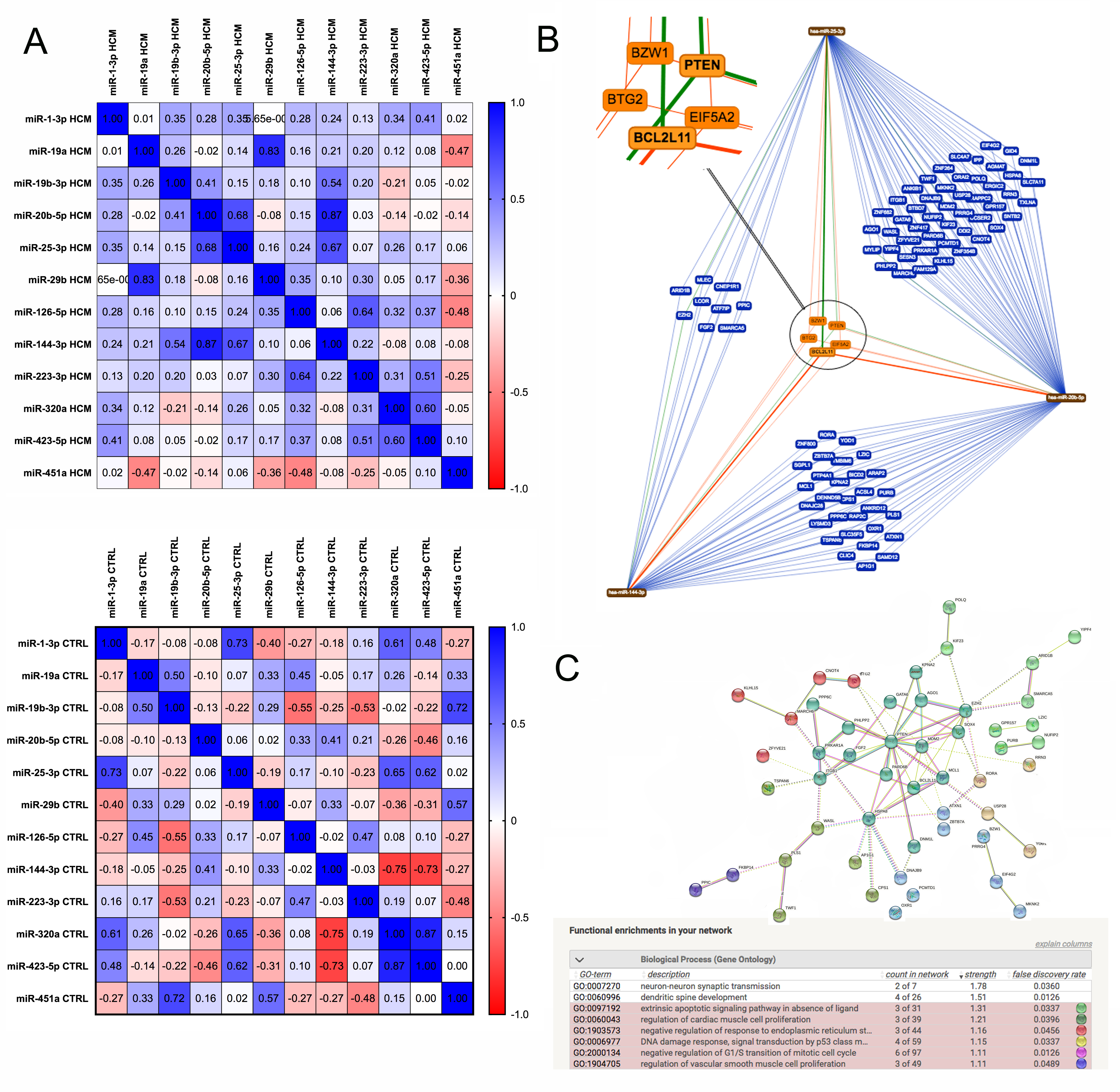


**Supplemental Figure 4 Top level plasma miRs by RT-qPCR: correlations**

Heatmaps show the correlation between miR pairs highly expressed into HCM and CTRL groups (i.e. the miRs with highest 2^-delta Ct absolute values in both groups) (**A**). Different colors corresponding to the same miR pairs in the 2 heatmaps visually highlight the inter-group differences. Spearman R values are displayed into heatmap cells. The complex network (by HumanTargetLink) connecting the hsa-miR-144-3p with hsa-miR-20b-5p and hsa-miR-25-3p and their putative target genes is shown (**B**). The corresponding protein network (by STRING v11) and the gene ontology analysis of related pathways is presented (**C**).

**­­­
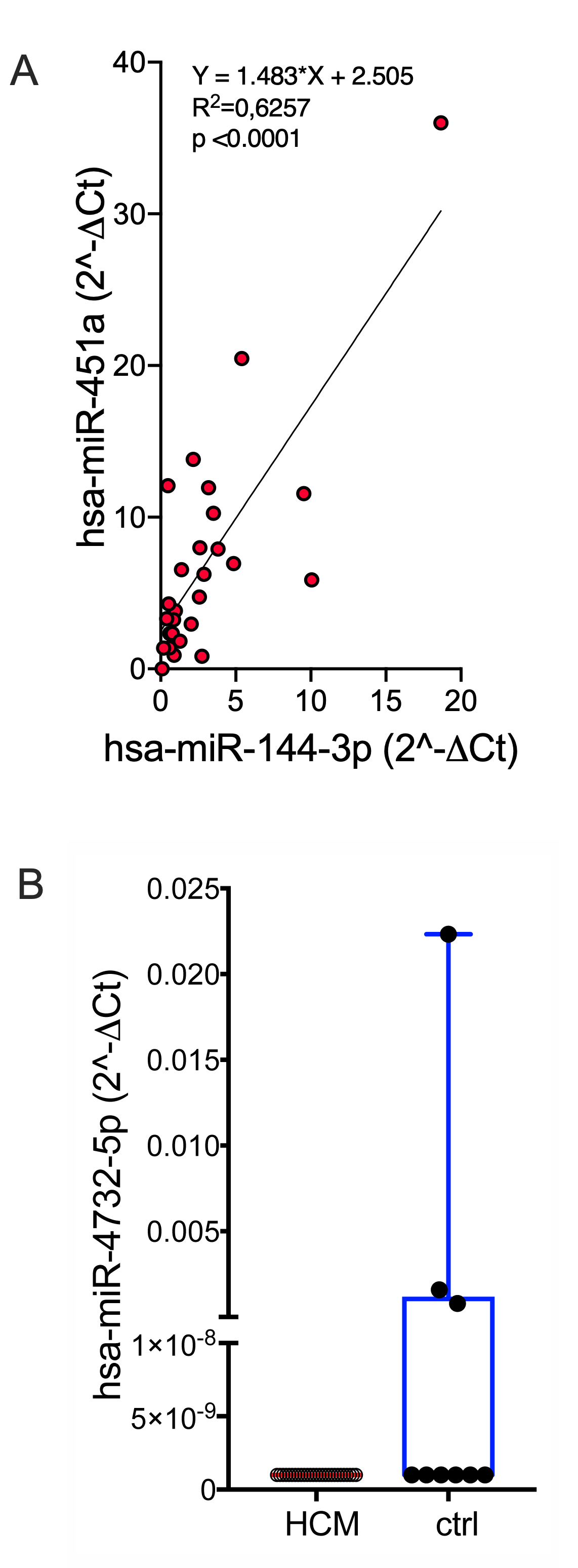
**

**Supplemental Figure 5 miR 144/451 cluster in myocardial tissue of HCM**

Linear relations between the hsa-miR-144-3p and hsa-miR-451a expression levels determined by RT-qPCR in HCM myocardial tissue are shown (**A**). Comparison between the expression levels of hsa-miR-4732-5p in HCM vs. ctrl myocardial tissues is plotted (**B**). Values are presented as boxes (min to max) and dots indicate single sample values.


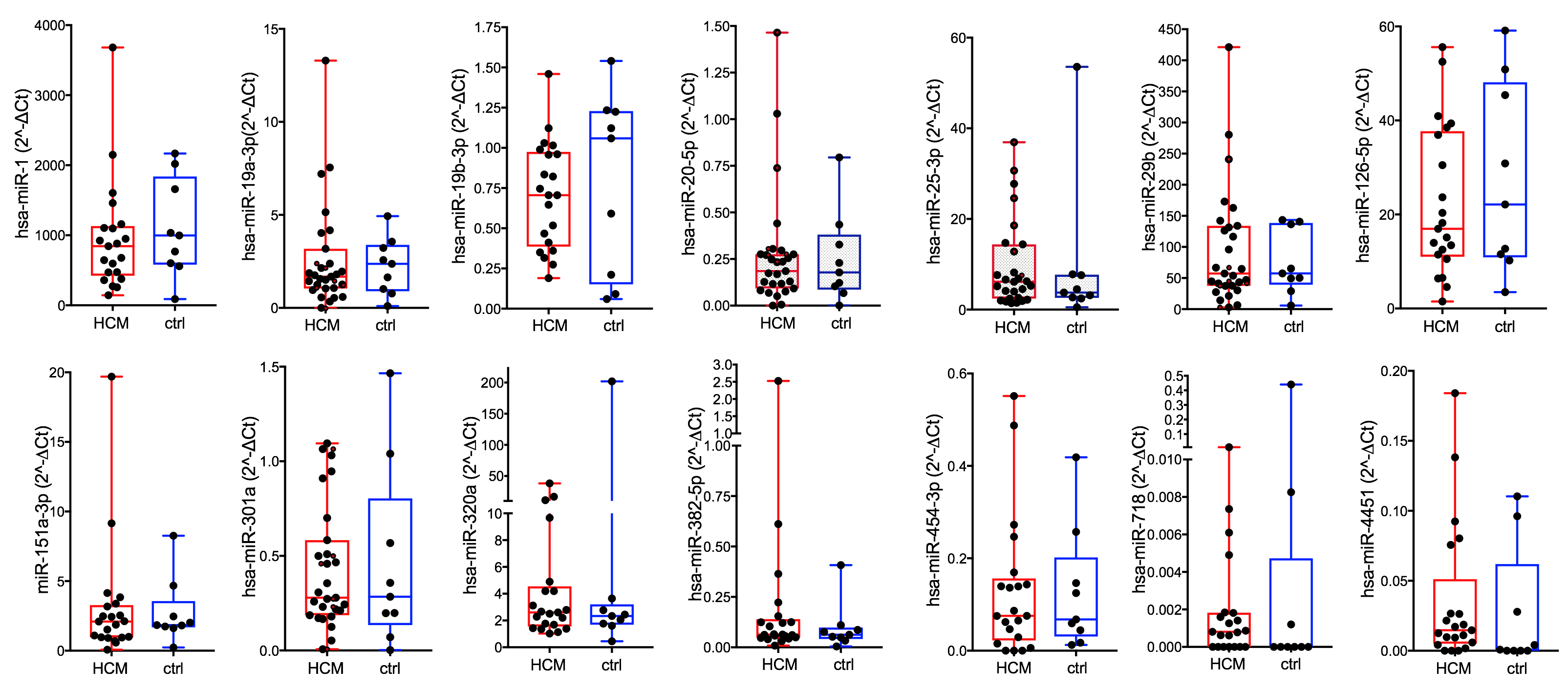


**Supplemental Figure 6 Analysis of tissue miRs by RT-qPCR**

RT-qPCR results on the expression levels of miRs in the myocardial tissues from HCM and ctrl showing not differentially expressed miRs is plotted. Values are presented as boxes (min to max) and dots indicate single sample values.


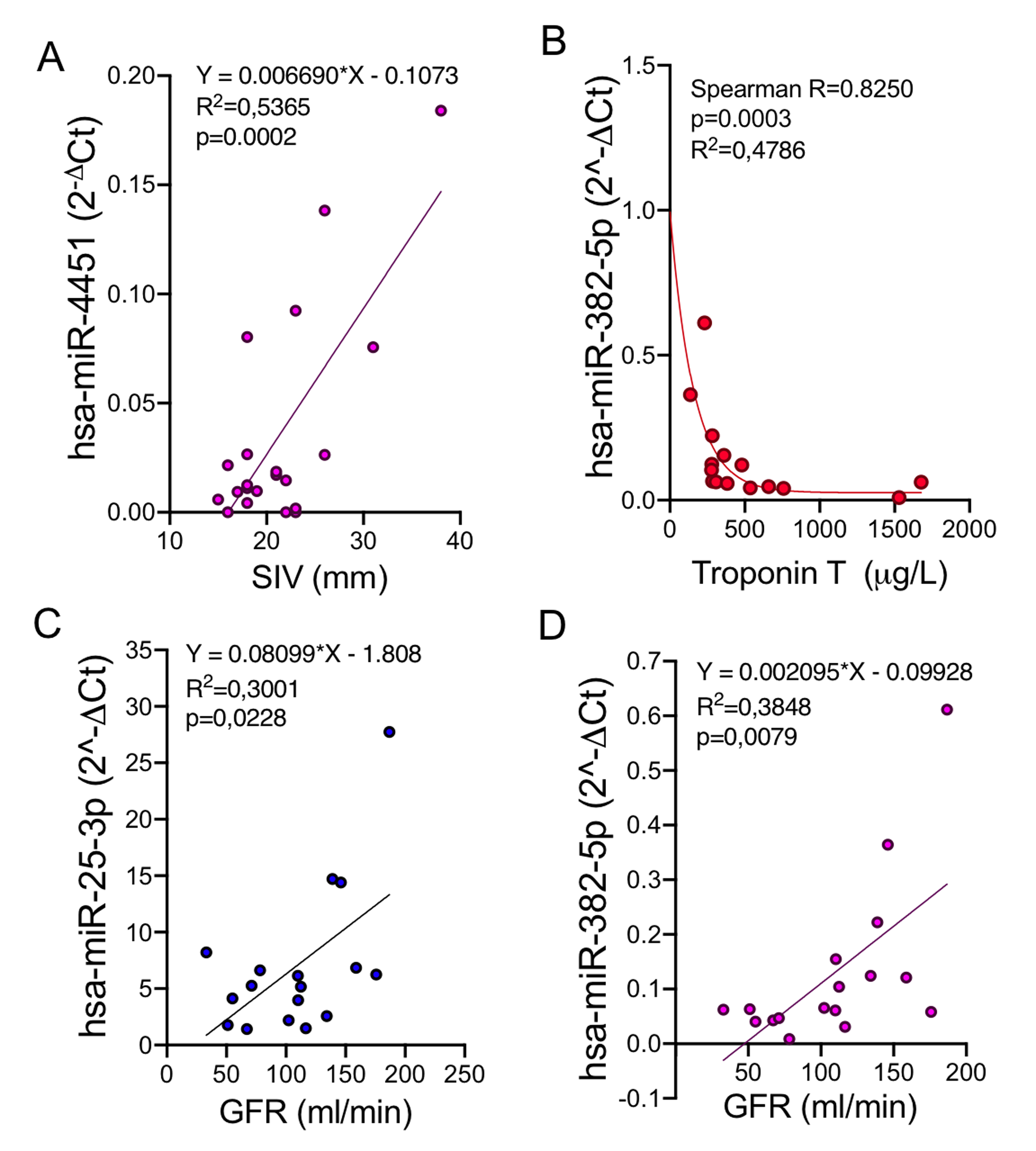


**Supplemental Figure 7 Myocardial miRs association with clinical data.** Linear relations between expression levels of 3 miRs, not differentially expressed in the myocardial tissues of HCM vs. ctrl (by RT-qPCR), and interventricular septum thickness (**A**), circulating Troponin T (**B**), or glomerular filtration rate (**C, D**) are plotted.


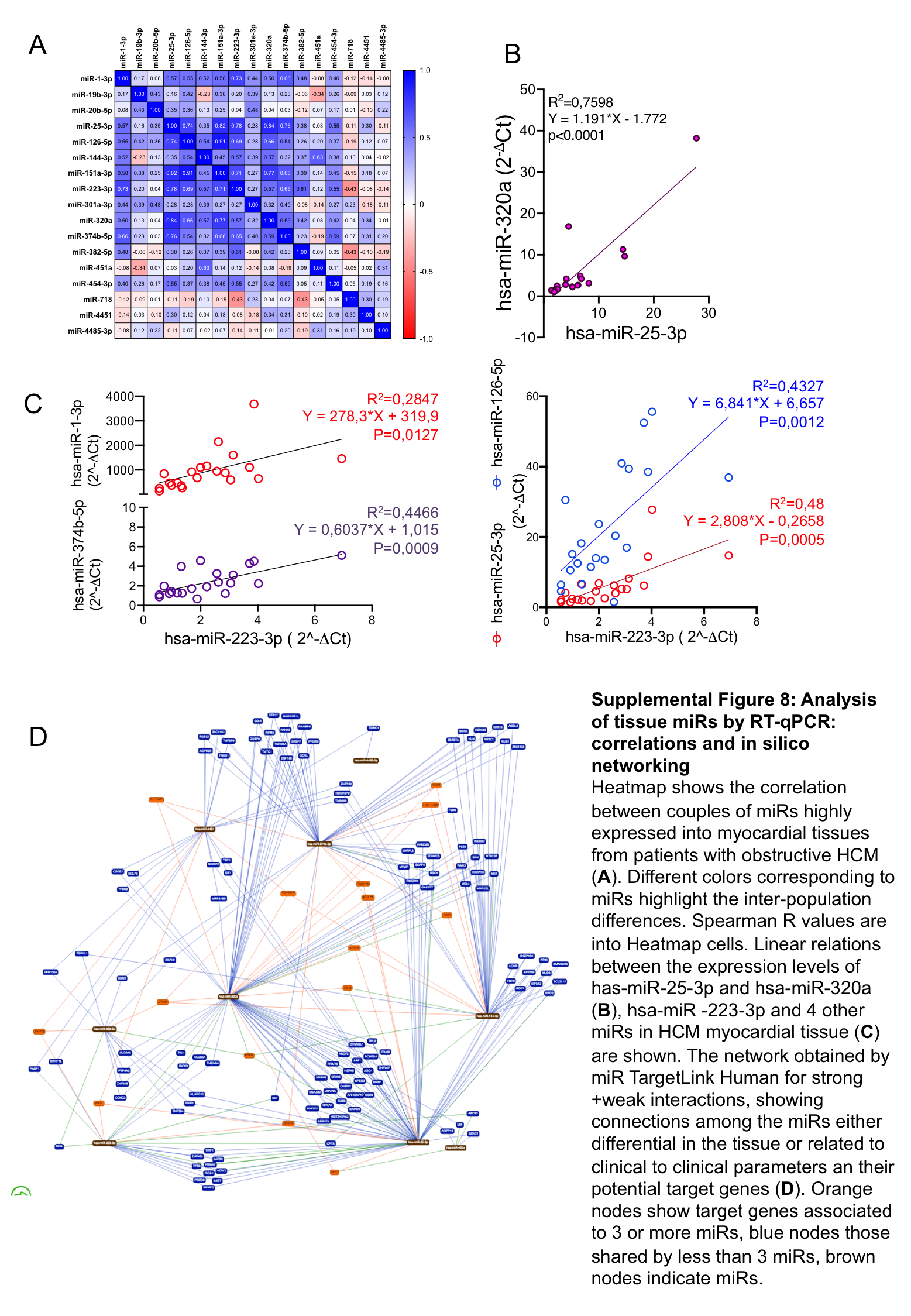

Supplement: Supplementary file 1 — Supporting Information [file CTM2-11-e435-s003.docx]
